# Modulation of volatile compound metabolome and transcriptome in grape berries exposed to sunlight under dry-hot climate

**DOI:** 10.1186/s12870-020-2268-y

**Published:** 2020-02-04

**Authors:** Lei He, Xiao-Qing Xu, Yu Wang, Wei-Kai Chen, Run-Ze Sun, Guo Cheng, Bin Liu, Wu Chen, Chang-Qing Duan, Jun Wang, Qiu-Hong Pan

**Affiliations:** 1 Center for Viticulture and Enology, College of Food Science and Nutritional Engineering, China Agricultural University, Beijing, 100083 China; 2 Key Laboratory of Viticulture and Enology, Ministry of Agriculture and Rural Affairs, Beijing, 100083 China; 3 Key Laboratory of Plant Resources, Institute of Botany, Chinese Academy of Science, Beijing, 100093 China; 4 CITIC Guoan Wine Co. Ltd., Manas, Xinjiang, 832200 China

**Keywords:** Leaf removal, Leaf moving, Half leaf removal, Volatile profiles, Grape berry

## Abstract

**Background:**

Basal leaf removal is widely practiced to increase grape cluster sunlight exposure that controls berry rot and improves quality. Studies on its influence on volatile compounds in grape berries have been performed mostly in Mediterranean or marine climate regions. It is uncertain whether similar efficiency can be achieved when grape berries are grown under continental climate. This study aimed to dissect the variation in volatile compound production and transcriptome in sunlight-exposed grape berries in a dry-hot climate region and to propose the key genes related to the variation.

**Results:**

Four cluster sunlight exposure strategies, including basal leaf removal at pepper-corn size stage, leaf removal at véraison (LR-V), leaf moving at véraison (LM-V), and half-leaf removal at véraison, were implemented at the north foot of the Mt. Tianshan region of northwestern China. Various cluster exposure treatments resulted in a decline in the concentrations of norisoprenoids and monoterpenes in ripening grape berries. Both *β*-carotene and lutein, the substrates of norisoprenoid biosynthesis, were reduced by cluster sunlight exposure. K-means cluster analysis showed that some genes involved in biosynthesis such as *VviTPS55*, *VviTPS60*, *VviTPS66*, *VviCCD4a* and *VviCCD4b* exhibited lower expression levels in exposed berries at least at one of the tested stages. Two C6-derived esters with fruity attributes, ethyl hexanoate and hexyl acetate, were reduced markedly. In contrast, main C6 alcohol compound levels were elevated in the LR-V- and LM-V-treated grape berries, which corresponded to the up-regulated expression of *VviLOXA*, *VviLOXO* and *VviADH1* in the oxylipin pathway. Most of the differentially expressed genes in the exposed and control berries were enriched to the “stress response” processes, and this transcriptome difference was accumulated as the berries matured. Besides, LR-V treatment stimulated a significant up-regulation in photosynthesis-related genes in the grape berries, which did not happen with LM-V treatment.

**Conclusions:**

Cluster sunlight exposure in dry-hot climate viticulture resulted in different volatile-targeted transcriptomic and metabolic responses from those obtained in the temperate Mediterranean or marine climate region. Therefore, a modified canopy management should be adopted to improve the aroma of grape berries.

## Background

Sunlight is one of the most important abiotic factors for plant growth and development. It can be converted to chemical energy, which is then used for synthesizing organic compounds via photosynthesis; altered sunlight conditions can exert a significant influence on the growth and chemical composition of grape berries [1]. Some canopy management practices such as leaf removal, cluster thinning, grapevine training, and leaf moving are widely used to optimize the canopy microclimate, allow varying sunlight exposure, control berry yield, and improve grape berry and wine quality [2]. Among these viticulture practices, leaf removal in a cluster zone (also called basal leaf removal) has been most commonly conducted, primarily because of its ability to promote sunlight exposure and airflow as well as to reduce foliage cover and disease incidence [3, 4]. It has also been found that artificial defoliation has a positive effect on phenolic and volatile compounds in grapes and wine [5, 6].

Leaf removal is generally performed in cool regions with appropriate sunshine and heat accumulation and rainfall [7]. It is typically conducted to selectively or completely strip the foliage from around the bunch zone, and this practice is traditionally implemented at a certain time after fruit set, usually before véraison [6, 8]. In the face of global warming combined with the sensitivity of grape berry ripening to climate change, viticulture management implemented in sunshine- and heat-appropriate regions should be adjusted to adapt to the warming climate [9]. In some strong sunshine and arid regions such as the wine producing regions in northwestern China, grapevine leaf removal in the green-fruit period occasionally causes grape berry sunburn and even leads to lignified and browned stems, which can cause the grape berries to stop growing due to nutrient deficiency. Moreover, the ripening progression of grape berries in this region is always accelerated due to the dry and hot climate [10, 11]. The shorted ripening duration also results in phenolic compound deficiencies, especially anthocyanins and phenolic co-pigments (e.g. myricetin, quercetin, catechin, epicatechin) that are sensitive to changes in climatic conditions and can compromise the color intensity and stability of wine [12]. Accordingly, it is necessary to adjust the timing of cluster sunlight exposure in dry-hot climate viticulture. Our previous study has shown that leaf removal or leaf moving at véraison, which exposes grape clusters to sunlight until harvest, can markedly improve the accumulation of flavon-3-ols and reduce the concentrations of anthocyanins in grape berries grown on the north foot of the Mt. Tianshan region of Xinjiang in northwestern China [5]. The aim of the present study was to dissect the variation in volatile compound metabolome and transcriptome in these exposed grape berries in this dry-hot climate region.

Grape-derived volatile compounds play the most role in evaluating the quality of grapes and wine. Previous studies have reported the effects of basal leaf removal at pre-véraison on the accumulation of monoterpenes and norisoprenoids that contribute to the Muscat varietal aroma and pleasant odor of grape [8, 13, 14]. Moreover, basal leaf removal causes variation in other volatile compounds such as methoxypyrazine [4, 15], thiol [16], and rotundone [17], which impart the vegetal, citrus, and black pepper aromas in grape berries. Indeed, the timing and intensity of sunlight exposure have distinct influences on the volatile compounds produced in grape berries. As Kwasniewski et al. observed [14], only cluster sunlight exposure starting at 33 days past berry set (PBS) significantly increases the concentration of total 1,1,6-trimethyl-1,2-dihydronaphthalene (TDN) and vitispirane, whereas leaf removal at 68 days PBS reduces *β*-damascenone generation. Additionally, when all basal leaves are removed to completely expose the grape cluster to sunlight, the berries accumulate more *β*-damascenone and some bound-form terpenoids [6]. Cluster sunlight exposure by apical defoliation approaches, compared with basal leaf removal, can minimally influence wine volatile compounds but reduce wine alcohol content [3]. A limited number of investigations have dealt with the change in volatile C6/C9 compounds in grape berries exposed to sunlight by leaf removal at the early stage of berry development [6, 18, 19]; however, the influence of leaf removal at the véraison or ripening stage has not yet been understood. The C6 aldehydes and alcohols can give rise to the characteristic ‘green’ odor, also called ‘green leaf volatiles’ (GLVs). These compounds are induced by the disruption of plant tissues or after plants suffer biotic or abiotic stresses [20]. C9 aldehydes, especially (*E*)-2-nonenal and (*E*,*Z*)-2,6 nonadienal, contribute to the cucumber flavor in plants [21]. Previous studies have also not dealt with the variation in volatile benzenoid-derived compounds in grape berries caused by leaf removal. Such compounds can confer floral and fruity flavors to grape berries and their corresponding wines [22, 23]. Understanding the variation in grape-derived volatile profile benefits an overall evaluation of how leaf removal in regions with intense sunshine and little rainfall will contribute to grape aroma quality improvement strategies.

Leaf removal may eliminate potential assimilated carbon supplements that the fruit receive from neighboring leaves, whereas leaf moving from around clusters enables vines to not only retain the photosynthetic organs but also increase the cluster sunlight exposure. Leaf removing at véraison could significantly promote the accumulation of total anthocyanins and up-regulate related genes [24], but the influence of this performance on the production of volatile compounds remains unclear. Furthermore, a previous transcriptomic study has only focused on the influence of cluster sunlight exposure at the early growth stage of grape berries (E-L 29) [8], whereas the transcriptomic response in grape berries to leaf removal or leaf moving at véraison or the ripening stage is poorly understood.

In this study, four cluster sunlight exposure strategies including leaf removal at the pepper-corn size stage (LR-PS), leaf removal at véraison (LR-V), half-leaf removal at véraison (HLR-V), and leaf moving at véraison (LM-V). A combined analysis of volatile metabolome and transcriptome data was conducted to elucidate the efficiency of these cluster sunlight exposure manipulations on grape berry volatile compound production, and the underlying mechanisms.

## Results

### Variation in cluster zone microclimate and berry physicochemical index by sunlight exposure

Unlike the temperate marine climate regions such as in Oregon, USA [5, 6], our experimental vineyard is characterized by a dry-hot desert climate with a total sunshine time of 2550–3500 h, precipitation of 90–100 mm, and evaporation of nearly 1000 mm in the grape growing season from April to September [5]. The distinct weather conditions indicate that similar cluster sunlight exposure treatments have different effects on the chemical composition and concentration of grape berries. We have previously described the variation in microclimate around the cluster zone, total soluble solids (TSS, ^o^Brix), and titratable acidity (TA) of grape berries following sun exposure [5]. At around véraison, the daily temperature around the berry clusters was slightly elevated by leaf removal (LR), half leaf removal (HLR), and leaf moving (LM) (Additional file 1: Figure S1). Moreover, these sunlight-exposure treatments also increased the mean hourly temperature of 1 day in the period from E-L 35 to E-L 36, by approximately 1.5 °C from 10:00 to 19:00. The daily air temperature ranged from 15.9 °C to 32.7 °C for the exposed clusters versus a range of 15.9 °C to 30.9 °C for the control. Correspondingly, altered exposure to sunlight markedly increased photosynthetically active radiation (PAR) and solar radiation (SR) around the berry cluster during development, as well as resulted in a reduction in relative humidity (RH) (Additional file 1: Figure S1). Unlike in the control, LR-PS and HLR-V treatment reduced total soluble solid (TSS) by about 0.73 and 1.70 ^o^Brix in the grape berries at ripening harvest, respectively, whereas both LM-V and LR-V treatments almost did not alter the TSS content of berries. Titratable acid (TA) content in the grape berries was also not altered by various sunlight exposure treatments (Additional file 2: Table S1).

### Variation in volatile compounds by cluster sunlight exposure

**Fig. 1:**
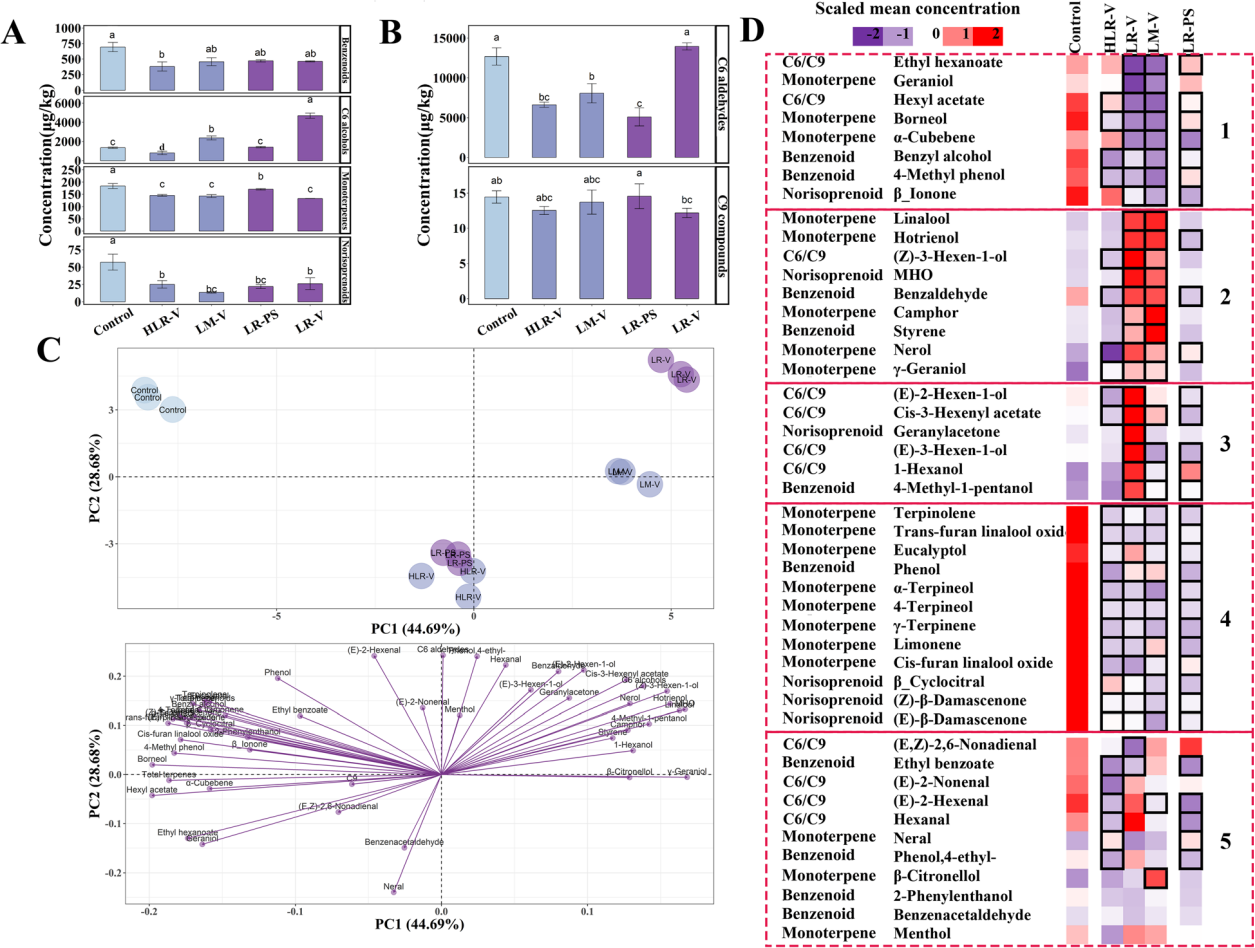
The effects of sunlight exposure treatments on volatile compounds. **a** Concentrations of free and glycosylated benzenoid, C6 alcohol, monoterpene and norisoprenoid in the exposed and control ripening berries. **b** Concentrations of free-form C6 aldehyde and C9 compounds in the exposed and control ripening berries. **c** Score plot and loading plot of principal components 1 and 2 for the measured variables. Different letters indicate significant differences (*P* = 0.05). **d** Hierarchical cluster analysis of all variables in the treatments and control. Volatile compounds are grouped into five clusters according to the responses to the treatments. Boxes with bold margins indicate significant differences (*P* = 0.05) between the treatment and control at ripening stage. HLR-V, half leaf removal at véraison; LM-V, leaf moving at véraison; LR-PS, leaf removal at berry pepper-corn size; LR-V, leaf removal at véraison

Free and glycosidically bound volatile compounds were separately determined. We found that the majority of C6 alcohols, benzenoids, norisoprenoids, and monoterpenes were present in both forms, but C6 aldehydes and C9 compounds were present only in the free form. To illustrate the effects of cluster sunlight exposure on the accumulation of volatile compounds, we decided to sum up the concentration of free and glycosidically bound forms of each compound, and the results are shown in Fig. 1. The results showed that only HLR-V treatment reduced the concentration of volatile benzenoids in comparison to the control, and other sunlight exposure treatments had no statistically significant effects on volatile benzenoids. The LM-V and LR-V treatments performed at véraison both significantly increased the concentrations of C6 alcohols, whereas HLR-V produced the opposite impact (Fig. 1a). It was also noticed that all the sunlight exposure treatments decreased the concentrations of total norisoprenoids and total monoterpenes in ripening grape berries (Fig. 1a). The other three exposure treatments, excluding LR-V, strongly suppressed the accumulation of C6 aldehydes, such as hexanal and (*E*)-2-hexenal, whereas the C9 compounds in the ripening grape berries were not altered with any of the sunlight exposure treatments tested (Fig. 1b).

Principal component analysis (PCA) was used to analyze the data of all volatile compounds from four treatments and the control with three biological replicates; the objective was to provide an overview of different cluster sunlight exposure effects on volatile compounds (Fig. 1c). The first (PC1) and second (PC2) principal components accounted for 73.37% of the total variance, with PC1 and PC2 explaining 44.69 and 28.68%, respectively. The control group with a high negative score for PC1 could be clearly separated from the LM-V and LR-V treatments with a high positive score for PC1. Both HLR-V and LR-PS treatments were concentrated on a negative half-axis of PC2 and close to the zero-axis of PC1, which were distinguishable from the control group by PC2. These findings indicated a relatively significant difference in volatile compound profiles among the control group, LM-V/LR-V group, and HLR-V/LR-PS group. However, the HLR-V and LR-PS sub-groups could not be clearly differentiated from each other, suggesting that they could have similar volatile profiles of ripening berries. Furthermore, there was also a certain difference between the two full cluster exposure treatments to sunlight at véraison: LR-V treatment was situated in a positive axis of PC2 with high score and LM-V treatment was close to the zero-axis of PC2. The corresponding loading plot reflected the relative importance of individual volatile compounds (Fig. 1c), and heatmap cluster analysis illustrated the change of each compound (Fig. 1d). The concentration of each volatile compound is shown in Table S2. It was found that some monoterpenes (e.g. linalool, hotrienol, nerol, and *γ*-geraniol) and benzenoids (benzaldehyde and styrene), together with 6-methyl-5-hepten-2-one (MHO) and (*Z*)-3-hexen-1-ol, were concentrated in the upper-right quadrant, and these components were present in relatively higher concentrations in the LR-V- and LM-V-treated berries, as shown in the cluster 2 of Fig. 1d. In contrast, most of the monoterpenes and norisoprenoids were located in the upper-left quadrant, corresponding to the site of the control group, which indicates that these compounds are present in higher levels than in the sunlight exposure treatment groups (cluster 1 and cluster 4 of Fig. 1d). In particular, the compounds shown in the cluster 1, such as ethyl hexanoate, geraniol, hexyl acetate, and *β*-ionone, were significantly lower than in the control group. Additionally, we also observed that the LR-V treatment had a positive effect on the accumulation of the compounds in cluster 3 (e.g. (*E*)-2-hexen-1-ol, (*E*)-3-hexen-1-ol, and 1-hexanol), which resulted in the higher concentration of total C6 alcohols in LR-V-treated berries (Fig. 1a). It should be noted that the concentrations of compounds in cluster 5 were increased or decreased in the exposed grape berries (Fig. 1d). It is thus difficult to explain whether the variation in these components was related to the cluster exposure to light.

**Fig. 2:**
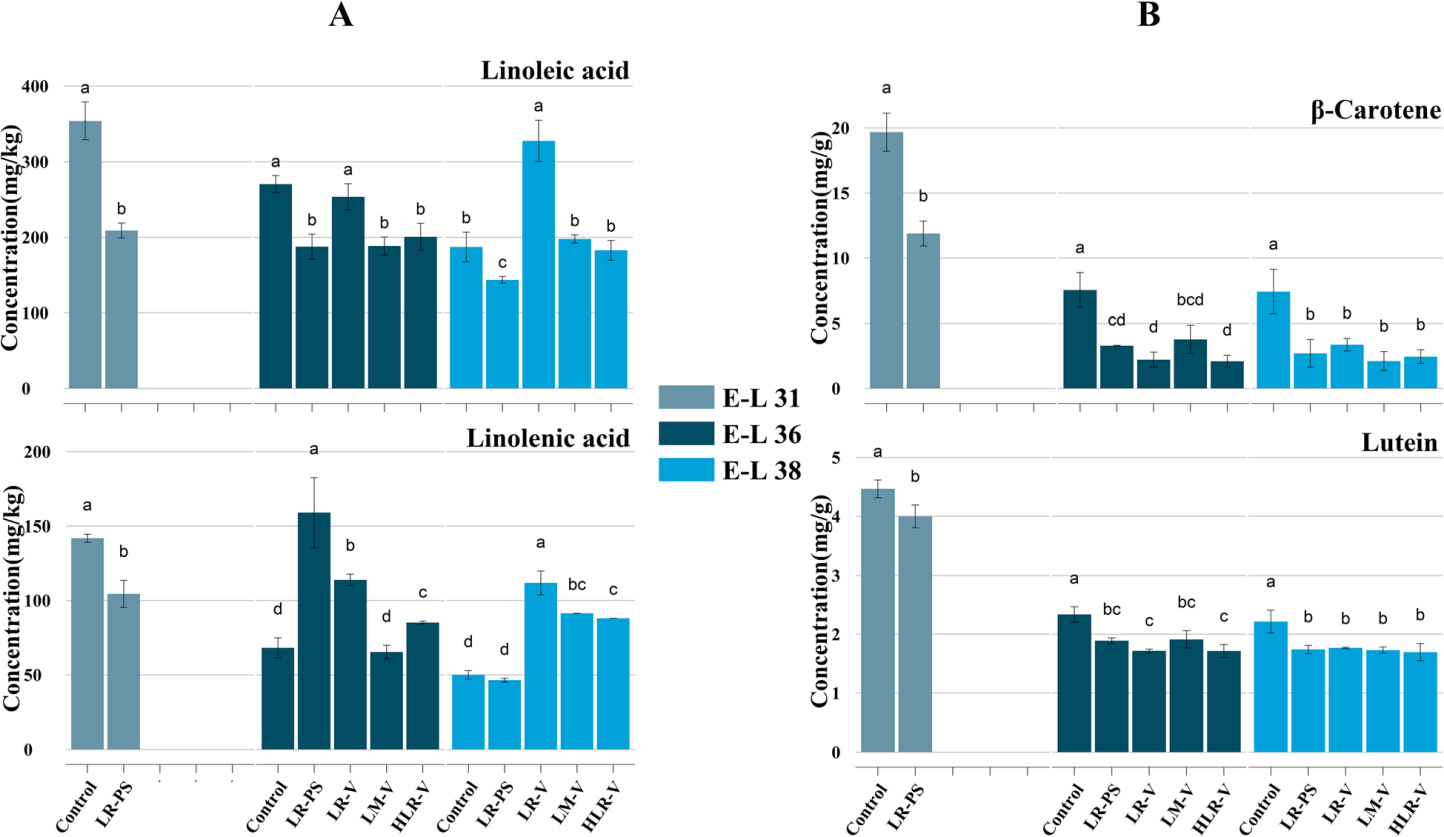
Changes of selected unsaturated fatty acids and carotenoids in the treated and control berries at E-L 31, E-L 36 and E-L 38 stages. **a** Concentration of linoleic acid and linolenic acid from the exposed and control berries. **b** Concentration of β-carotene and lutein from the exposed and control berries. Different letters indicate significant differences (*P* = 0.05). HLR-V, half leaf removal at véraison; LM-V, leaf moving at véraison; LR-PS, leaf removal at berry pepper-corn size; LR-V, leaf removal at véraison

Two types of volatile precursors were examined in mature berries. Through the lipoxygenase (LOX)-hydroperoxide lyase (HPL) pathway, linoleic acid can be cleaved to generate hexanal, hexanol, and their derivatives, whereas linolenic acid can be converted into hexenal, hexenol, and their derivatives [25]. In this study, the concentration of linoleic acid was substantially elevated by LR-V treatment at E-L 38 (ripening harvest) and of linolenic acid at E-L 36 and E-L 38 stages (Fig. 2a), which is agreement with the significant increase in most C6 compounds with LR-V treatment (see cluster 3 of Fig. 1d and hexanal in cluster 5). *β*-Carotene and lutein are two important carotenoids in grape berries that can be cleaved to generate norisoprenoids via CCDs. It was found that the concentrations of the two precursors were reduced in all sunlight-exposed grape berries at E-L 31, E-L 36, and E-L 38 stages (Fig. 2b). Combined with the declining norisoprenoid concentration (Fig. 1a), it can be inferred that cluster exposure to sunlight could cause an overall down-regulation of norisoprenoid biosynthesis.

### Transcriptional reprogramming by LR-V and LM-V treatments

**Fig. 3:**
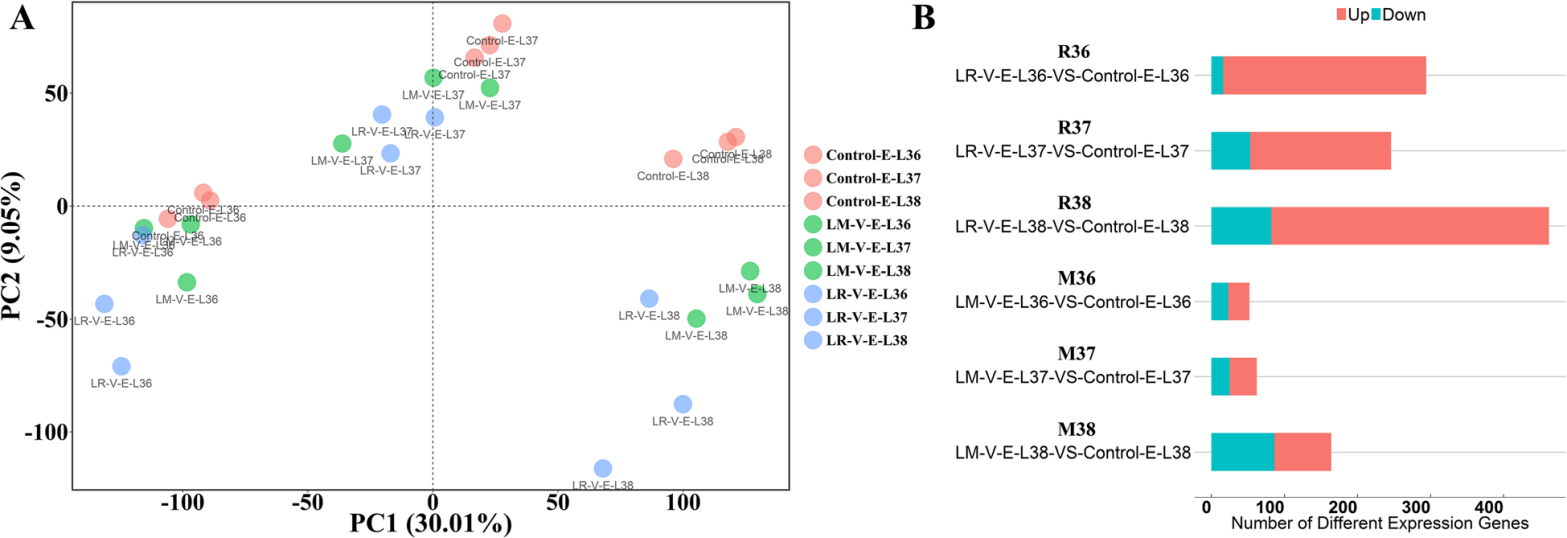
Transcriptional response to the sunlight exposure treatments. **a** Principal component analysis of the group of treatment and control berries at three ripening stages. The quantitative variables correspond to transcript abundance of 28,940 grape genes. Each circle represents a biological replicate. **b** Comparison of number of differentially expressed genes between different samples. Red bars and cyan bars, respectively, represent the number of up-regulated and down-regulated genes in the treatments of LR or LM in relative to the control samples at the certain stage

Based on the above results, LR-V and LM-V treatments were demonstrated to markedly alter volatile compound profiling as well as the levels of some volatile precursors detected in this study. To explain the variation in volatile compounds due to cluster sunlight exposure at véraison, from the view of transcriptome, we performed RNA-sequencing for LR-V, LM-V and the control grape berries at the E-L 36, E-L 37 and E-L 38 stages in the mature period. In total, 28,940 genes were annotated, and then used for PCA analysis, based on their expression levels. The control group could not be clearly differentiated from the LV-R and LM-R groups at the E-L 36 stage (Fig. 3a), suggesting that the difference in gene expression profiles at these stages are limited. However, the transcriptomic difference was gradually increased as the berries matured. The LM-V-E-L 38 and LR-V-E-L 38 treatments could be distinguished by PC2 from the control-E-L 38 (Fig. 3a). This indicates that the LM-V or LR-V treatment exerted a cumulative effect on the transcriptome of grape berries. The results were also demonstrated by comparing the number of differentially expressed genes (DEGs). The DEGs were selected from the following six comparison sets: LR-V-vs-Control and LM-V-vs-Control at E-L 36 (abbreviated as R36 and M36), E-L 37 (abbreviated as R37 and M37) and E-L 38 (abbreviated as R38 and M38), respectively. The R38 comparison generated the most DEGs among 3 DEG sets that related to LR-V-vs-Control (E-L 36, E-L 37 and E-L 38). Similarly, M38 also had the largest number of DEGs among the three sets that corresponded to the LM-V-vs-Control comparisons at the three developmental stages (Fig. 3b). Moreover, there were more DEGs in the LR-V-vs-Control sets (R36, R37 and R38) than in the LM-V-vs-Control of the three developmental stages (that is, M36, M37 and M38). This was possibly because the LR-V treatment resulted in the loss of vegetative organs around the grape cluster in addition to improving sunlight exposure. As a consequence, grape berries had to undergo multiple changes to adapt themselves to their new growth conditions.

**Fig. 4:**
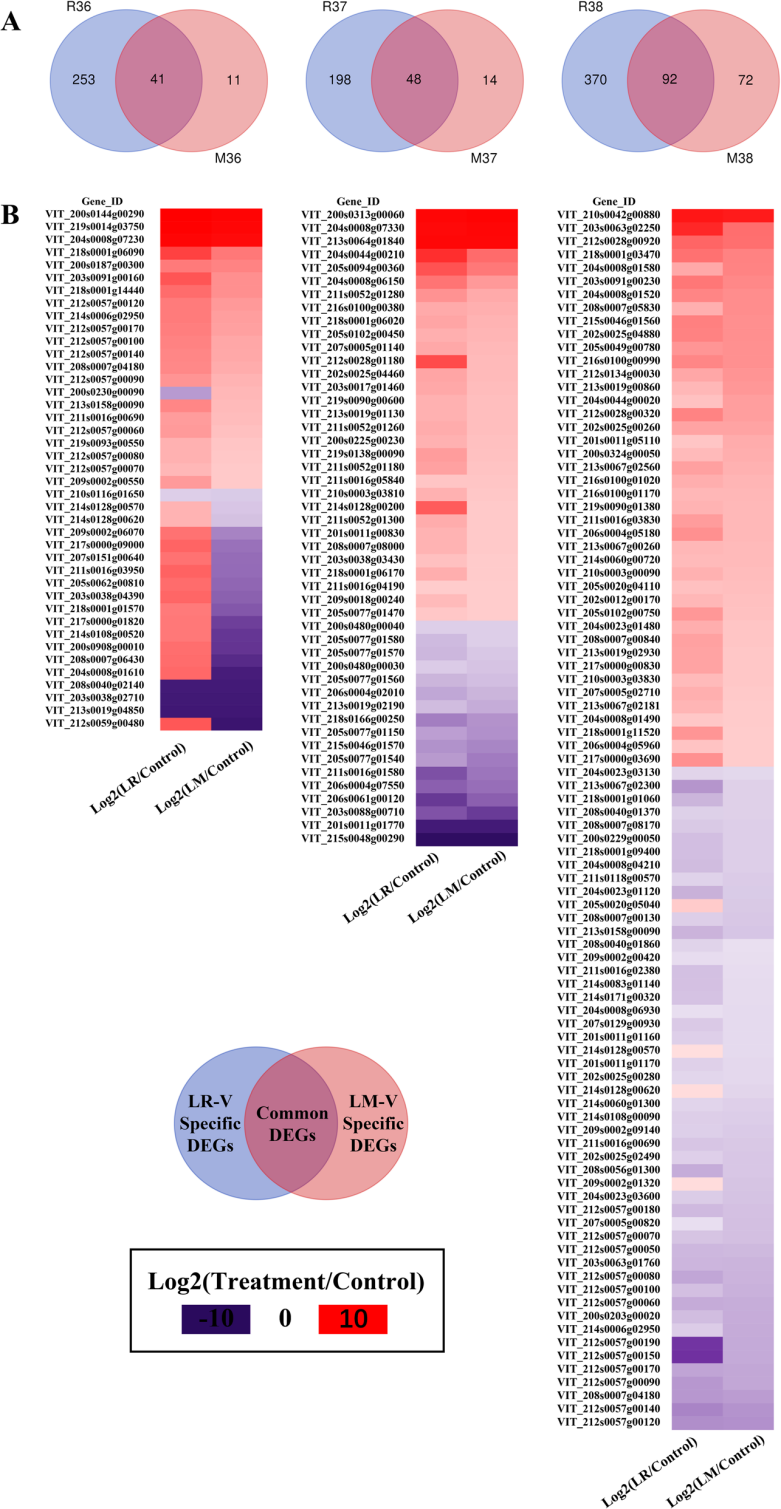
Similarities and differences of DEGs by LM-V and LR-V. **a** Venn diagram displaying common and unique DEGs when comparing the two treatments to the control. The 6 DEG sets of R36, M36, R37, M37, R38 and M38 corresponding to the comparison of LR-V-VS-Control and LM-V-VS-Control at E-L 36, E-L 37 and E-L 38, respectively. **b** Hierarchical cluster analysis of common DEGs induced by both LM-V and LR-V. Purple and red boxes indicate downregulated and upregulated genes, the colors of the boxes represent the intensity of the expression fold changes (log2)

Venn diagrams using a heatmap were constructed to identify and explore the common and specific DEGs of LR-V-vs-Control and LM-V-vs-Control comparisons at the same developmental stage. As shown in Fig. 4a, there were 41, 48, and 92 common DEGs at E-L 36, E-L 37 and E-L 38, respectively, suggesting that these common DEGs should be closely related to the response of the grape berries to sunlight exposure. However, there were 253, 198, and 370 DEGs that were specific to the R36, R37 and R38, whereas 11, 14, and 72 DEGs uniquely appeared in the M36, M37 and M38. Figure 4b illustrates that the majority of common DEGs had similarly up- or down-regulated expression patterns in the leaf removal (LR) and leaf moving (LM) treatments. Their functional annotation further indicated that most of the common DEGs were involved in grape berry ripening and stress response (Additional file 4: Table S3). For example, four genes encoding xyloglucan endotransglucosylase/hydrolases (VIT_211s0052g01280, VIT_211s0052g01260, VIT_211s0052g01180, and VIT_211s0052g01300) were all up-regulated at E-L 37. Moreover, there were five pathogenesis-related genes (VIT_205s0077g01580, VIT_205s0077g01570, VIT_205s0077g01560, VIT_205s0077g01540, VIT_203s0088g00710) that were largely down-regulated at E-L 37. At the E-L 38 stage, several genes encoding small heat stress proteins (sHSPs) and stilbene synthase (STS) were also found to be commonly up-regulated. It has been widely known that the sHSPs are always correlated with plant abiotic stress tolerance [26], and the up-regulation of *VviSTS* expression may promote substrate utilization of *p*-coumaryl-CoA and malonyl-CoA [27] in grape berries and increase the generation of stilbenes. Considerable evidence has supported that stilbenes can be largely induced in plants subjected to biotic and abiotic stimuli [28, 29]. It is thus considered that the up-regulation of these genes encoding sHSPs and STS may be a consequence of grape berry response to increased sunshine. Nevertheless, some common DEGs related to biotic and abiotic stimuli at E-L 36 were down-regulated in the LM-V and LR-V berries, for example, genes that encode late embryogenesis abundant proteins (VIT_203s0038g04390, VIT_209s0002g06070 and VIT_200s0908g00010), small heat-shock proteins (VIT_218s0001g01570 and VIT_204s0008g01610), and germin-like protein (VIT_214s0128g00570 and VIT_214s0128g00620). Previous reports have remarked that late embryogenesis abundant protein (LEAP) is often associated with salt and drought stress tolerance in some plants [30, 31], and the germin-link protein (GLP) responds to both biotic and abiotic stress [32]. Meanwhile, we also observed that 16 genes showed the opposite responses to LR-V and LM-V at E-L 36, and most of them were up-regulated with LR-V treatment and down-regulated with LM-V treatment (Fig. 4b). Among the 16 DEGs, except for four genes encoding hypothetical proteins (VIT_212s0059g00480, VIT_200s0230g00090, VIT_214s0128g00620 and VIT_205s0062g00810), the others were mostly stress-related proteins, such as late embryogenesis abundant proteins (VIT_203s0038g04390, VIT_209s0002g06070 and VIT_200s0908g00010), HSP20 family proteins (VIT_218s0001g01570 and VIT_204s0008g01610) and dehydration-responsive protein rd22 (VIT_211s0016g03950). These stress response proteins, together with the genes encoding a malate synthase (VIT_217s0000g01820) and a nonspecific lipid-transfer protein (VIT_214s0108g00520), were all up-regulated with LR-V treatment but down-regulated with LM-V at E-L 36, compared to that with control treatment.

**Fig. 5:**
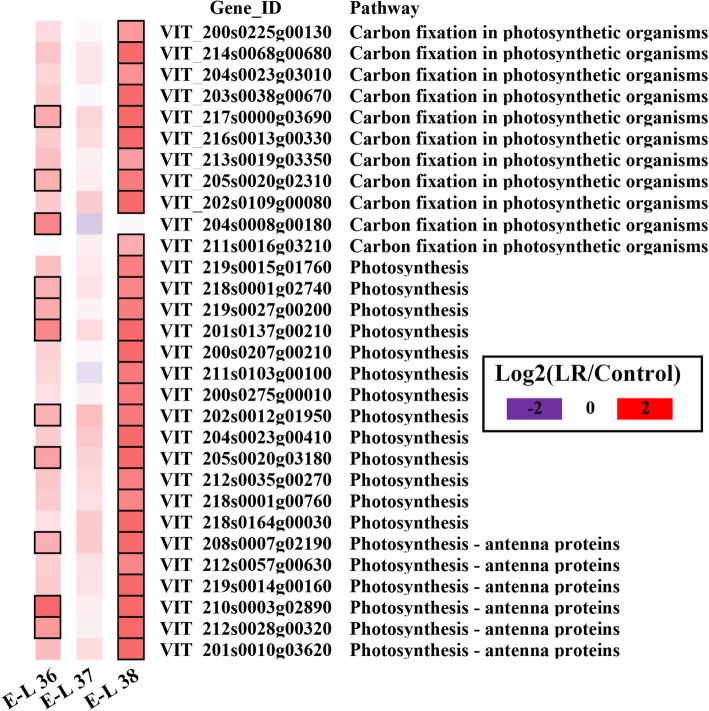
The selected DEGs only induced by LR-V. Purple and red boxes indicate down-regulated and up-regulated genes, the colors of the boxes represent the intensity of the expression fold changes (log2). Boxes with bold margins indicate significant differences (*P* = 0.05) between the treatment and control at ripening stage

To understand the metabolisms associated with the specific DEGs in the Venn diagrams (Fig. 4a), we then conducted KEGG pathway enrichment analysis. The DEGs specific to LR-V treatment were highly enriched in carbon fixation pathway, and were mainly photosynthesis-related proteins (Additional file 5: Table S4). Interestingly, these photosynthesis-related genes were significantly up-regulated in the LR-V treated grape berries, in particular at the E-L 38 stage (Fig. 5), although grape berries are not an important for photosynthesis. Perhaps the grape berry transcriptome reprograming was as a response to the lack of photosynthetic organs (that is, functional leaves) around them, but the biological effect of this variation still needs to be proved. Compared to the number of DEGs specific to LR-V, there were fewer DEGs specific to the LM-V treatment (see Fig. 4a). The LM-V specific DEGs were mainly enriched in the pathways associated with the synthesis of phenolic compounds (e.g. stilbenoid, diarylheptanoid and gingerol, flavone and flavonol, and flavonoids) and plant-pathogen interactions (Additional file 6: Table S5).

To further compare the grape berry transcriptome variation by the two cluster sunlight exposure treatments, we also identified DEGs between LM-V and LR-V. There were a total of 144, 111, and 284 DEGs at E-L 36, E-L 37 and E-L 38 stages, respectively (Additional file 7: Table S6). We noticed that about 90% of these DEGs were up-regulated with LR-V in comparison with LM-V at each stage, suggesting that sunlight exposure by leaf removal could lead to a complicated grape berry transcriptional response, in comparison with leaf moving. A total of 72 genes were up-regulated with LR-V and LM-V at all three sampling stages, including genes encoding one MADS-box transcription factor, one aquaporin TIP3–2-like, two HSP20 family proteins, one malate synthase and a number of stress-related proteins.

### Variation of volatile compound biosynthesis-associated gene expression profiles by cluster sunlight exposure

The alteration in volatile compound biosynthesis-associated gene expression profiles by cluster sunlight exposure at véraison was particularly assessed with the intent of interpretation on the changes in volatile compounds (Fig. 1). Monoterpenes are biosynthesized through two separate but tightly connected pathways: the plastidial 2-methyl-D-erythritol-4-phosphate phosphate (MEP) and cytoplasmic mevalonic acid (MVA) pathways [33]. To better illustrate genes that are mainly responsible for the accumulation of the volatile compounds evaluated in this study, some genes with low expression levels (RPKM ≤1) were omitted in the following analyses. It was found that genes in the MVA and MEP pathways were not significantly altered in the LM-V- and LR-V-treated grape berries (Additional file 1: Figure S2). The expression of *VviDXS*, annotated as *VviDXS1* (VIT_205s0020g02130), was not significantly altered at a transcriptional level; this gene has been identified as a critical quantitative trait locus (QTL) for monoterpenes concentration [34, 35]. Terpenoid synthase (TPS) family is composed of four subfamilies of *VviTPS-a, VviTPS-b, VviTPS-e and VviTPS-g*, among which both TPS-b and TPS-g subfamilies are characterized as monoterpene synthases, while the TPS-a subfamily is responsible for synthesis of sesquiterpenes [36]. Most of the genes encoding TPSs were excluded from this analysis due to very low expression abundance (RPKM < 1), and there were only 14 *VviTPSs* with an RPKM ≥1, (Additional file 1: Figure S2). Among them, only a TPS-b family gene *VviTPS35* (VIT_212s0134g00030) was markedly up-regulated by both LM-V and LR-V at the E-L 38 stage, whereas five TPS-g family genes showed insignificant up-regulation or fluctuation in response to the treatments. In grape berries, large amounts of monoterpenes are present as nonvolatile glycosides. These glycosides are formed by the action of monoterpene glycosyltransferases (GT), three of which have been functionally characterized [13, 37]. In this study, the genes encoding GT7, GT14 and GT15 were not significantly affected by LM-V and LR-V treatments.

The geranylgeranyl-diphosphate derived from the MEP pathway acts as the substrate for phytoene synthesis, which is catalyzed by phytoene synthase (PSY). Phytoene is then converted to generate a series of carotenoids that can be further cleaved into norisoprenoids by carotenoid cleavage dioxygenases (CCDs), or into abscisic acid, strigolactone, and other products by a series of enzymes [38]. It has been known that *VviCCD4a* and *VviCCD4b* are primarily responsible for the cleavage of carotenoids into norisoprenoids in developing grape berries [39]. In the present study, *VviCCD4a* and *VviCCD4b* expression was down-regulated in sunlight-exposed berries by the LR-V or LM-V treatment, but the expression was not statistically significant. In contrast, *VviNCED3*, which is strongly associated with the biosynthesis of endogenous ABA [38], was obviously up-regulated in the sunlight-exposed berries at the E-L 36 stage.

The C6 aldehydes, C6 alcohols, and volatile C9 compounds are synthesized all through the lipoxygenase–hydroperoxide lyase (LOX–HPL) pathway, in which lipoxygenase (LOX), hydroperoxide lyase (HPL), and alcohol dehydrogenase (ADH) are critical enzymes [40–42]. In total, eight *VviLOX*, one *VviHPL*, and six *VviADH* were identified in the present RNA sequencing analysis (Additional file 1: Figure S3). As one of the putative 13 LOXs, *VviLOXA* (VIT_206s0004g01510) is the most abundant and is primarily expressed during grape berry development [40]. However, the expression of this gene was not significantly altered by the LM-V and LR-V treatments in the present study. ADH is responsible for the conversion of aldehydes into alcohols, and a previous study found that the expression of *VviADH*2 parallels to ADH enzyme activity [41]. However, at present, *VviADH2* (VIT_204s0044g01110) was down-regulated in LR-V-treated berries at the E-L 38 stage, which did not correspond to an increase in C6 alcohols. Conversely, *VviADH*1 had higher expression abundance in the sunlight-exposed grape berries than in the control, specifically at the E-L 38 stage, suggesting that the expression of this gene may be closely related to the increase in C6 alcohols in LM-V- and LR-V-treated berries. Another branch pathway involves the biosynthesis of jasmonic acid (JA), which shares the 13-hydroperoxy linoleic acid (or linolenic acid) substrate with the C6 compound synthetic pathway driven by HPL. In this study, six genes encoding 12-oxo-phytodienoic acid reductase (OPDA) in the JA biosynthetic pathway were found to be up-regulated by the LM-V and LR-V treatments, especially *VviOPDA* (VIT_218s0041g02060). It has been reported that OPDAs are induced by biotic and abiotic stress accompanied by the formation of galactolipids esterified in *Arabidopsis thaliana* [43]. Due to a lack of JA concentration data, we could not determine whether this JA synthesis was activated in response to sunlight exposure. However, we concluded that *VviOPDA* (VIT_218s0041g02060) was strongly induced at the transcriptional level in the sunlight-exposed berries.

Most of volatile benzenoids are generated from phenylalanine and *trans*-cinamate, as shown in Additional file 1: Figure S4. Phenylacetaldehyde and phenylethylalcohol can be directly synthesized from phenylalanine when catalyzed by tyrosine/DOPA decarboxylase 1-like (TYDC) and primary amine oxidase (PAO). In this study, one *VviTAT* (VIT_219s0014g02190) and one *VviPAL* (VIT_200s2849g00010) were up-regulated by LM-V at the E-L 38 stage (Additional file 1: Figure S4). Furthermore, the *Vvi4HPPD* was up-regulated by LR-V treatment. These genes may be associated with the production of both benzenoids and flavonoids as they share the common substrate phenylalanine.

**Fig. 6:**
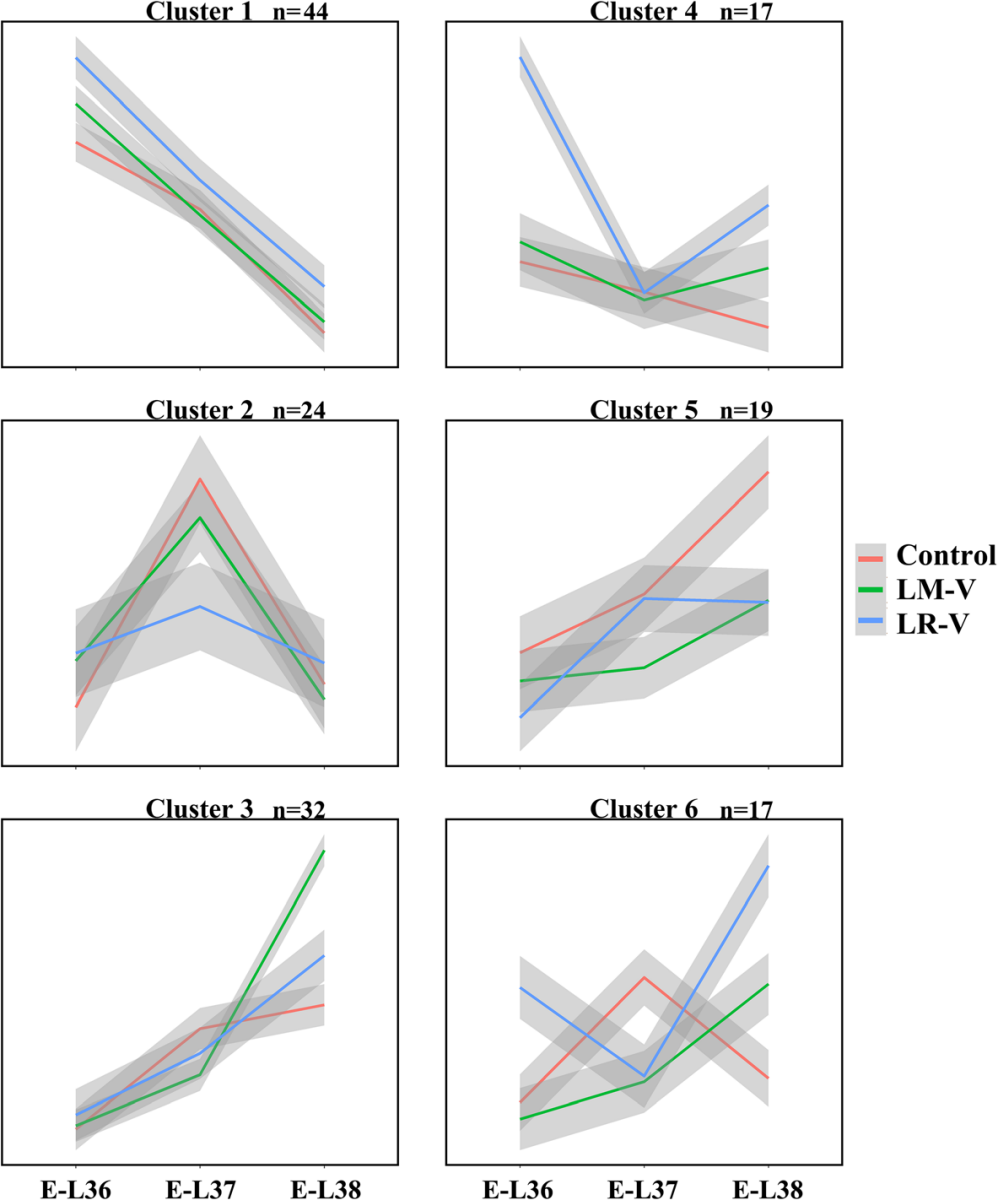
K-means cluster analysis of the time series for 153 genes involved in the biosynthesis of volatile compounds

As mentioned above, volatile compounds were affected substantially, but a majority of genes related to their biosynthesis were not significantly varied by the sunlight exposure treatments. It seems that DEG analysis cannot completely explain the difference in the corresponding metabolites. To understand whether sunlight exposure treatments has a synergistic effect on the expression of genes related to targeted volatile compound biosynthesis, we conducted k-means cluster analysis of the time series for volatile compound biosynthesis-related genes to investigate the gene expression pattern. R package ‘factoextra’ was used to determine the optimal number of clusters and six clusters were generated (Fig. 6). Cluster 1 was defined by a decrease in transcript accumulation from E-L 36 to E-L 38. Genes in cluster 1 showed a higher expression in LR-V-treated grape berries at E-L 36, mainly including 2 *VviADH*s, 3 *VviLOX*s, 4 *VviOPDA*s, 4 *VviPAL*s, 3*VviNCEDs* and some upstream genes of terpenoid and carotenoid metabolism, which corresponded to the increase in C6 alcohols (Table 1). In contrast, 24 and 19 genes exhibited higher transcript abundance in the control group in cluster 2 (E-L 37) and cluster 5, respectively, in which key genes for biosynthesis of monoterpenes and norisoprenoids such as *VviTPS55*, *VviTPS60*, *VviTPS66*, *VviCCD4a*, *VviCCD4b* were included. The 32 genes in cluster 3 were expressed at a higher level in LM-V-treated berries at E-L 38, comprising *VviTPS-a*, *VviTPS-b*, *VviTPS-e*, and genes related to methyl jasmonate biosynthesis. Furthermore, 34 genes presented higher levels in LR-V-treated grape berries at E-L 36 and E-L 38 in cluster 4 and 6. We found the other 2 *VviADH*s and 2 *VviLOX*s in this two clusters, which can also contribute to higher levels of C6 alcohols in exposed berries.

**Table 1:** Biosynthesis of volatile compound related genes in each cluster identified by k-means cluster analysis

Cluster	Gene		
	Linolenic acid metabolism	Phenylalanine metabolism	Terpenoid and carotenoid metabolism
1	ADH2(VIT_204s0044g01110), ADH1(VIT_218s0001g15410), LOXA (VIT_206s0004g01510), LOXO (VIT_209s0002g01080), LOX (VIT_206s0004g01450), OPDA (VIT_218s0041g02010), OPDA (VIT_218s0041g02070), OPDA (VIT_218s0041g02020), OPDA (VIT_218s0041g02060), OPCL1(VIT_218s0001g00290), OPCL1(VIT_217s0000g01790), ACX (VIT_208s0032g01100), ACAT (VIT_205s0051g00720)	CAAT (VIT_208s0058g01000), PAL (VIT_216s0039g01100), PAL (VIT_216s0039g01110), PAL (VIT_216s0039g01120), PAL (VIT_216s0039g01130), PAO (VIT_200s1937g00010), TAT (VIT_212s0028g03210)	ACCAT (VIT_218s0089g00520), ACCAT (VIT_218s0089g00570), ACCAT (VIT_218s0089g00590), HMGCR (VIT_218s0122g00610), HMGCR (VIT_204s0044g01740), HMGCS (VIT_202s0025g04580), MK (VIT_214s0128g00330), PMK (VIT_202s0012g02530), DXS1(VIT_205s0020g02130), DXS (VIT_200s0218g00110), MCT (VIT_212s0035g01950), MDS (VIT_202s0025g00370), IPPI (VIT_204s0023g00600), TPS (VIT_200s0271g00030), GT14(VIT_218s0001g06060), GT15(VIT_206s0004g05780), PSY1(VIT_204s0079g00680), PSY2(VIT_212s0028g00960), GGPPS (VIT_219s0090g00530), GGPPS (VIT_218s0001g12000), PDS (VIT_209s0002g00100), ZDS (VIT_214s0030g01740), LUT1(VIT_208s0007g04530), NCED3(VIT_219s0093g00550), NCED2(VIT_210s0003g03750), NCED (VIT_204s0008g03380), XDH (VIT_213s0019g01010), AAO (VIT_206s0009g00770)
2	ADH (VIT_204s0044g01130), LOX (VIT_206s0004g01480), LOX (VIT_201s0010g02750), HPL (VIT_212s0059g01060), OPDA (VIT_218s0041g02260), ACX (VIT_216s0022g01150)	CAAT (VIT_211s0016g03720), PAO (VIT_200s0225g00090)	HMGCS (VIT_214s0036g00810), PPMD (VIT_213s0106g00790), DXS (VIT_211s0052g01730), IPPI (VIT_214s0006g01710), IPPI (VIT_211s0206g00020), GPPS (VIT_215s0024g00850), TPS60(VIT_200s0385g00020), TPS26(VIT_219s0014g04810), TPS66(VIT_200s0372g00030), TPS55(VIT_200s0271g00010), CRTISO (VIT_208s0032g00800), NCED1(VIT_205s0051g00670), CCD4b(VIT_202s0087g00930), AAO (VIT_218s0041g02400), AAO (VIT_218s0041g02430),
3	AOS (VIT_218s0001g11630), ACX (VIT_200s0662g00010), ACX (VIT_212s0028g02660), ACX (VIT_208s0105g00460), ACX (VIT_216s0022g01120), ACX (VIT_211s0037g01380), MEP2(VIT_211s0016g03690), MEP2(VIT_205s0077g02140)	HPA (VIT_206s0004g02170), PAO (VIT_217s0000g09100), TAT (VIT_219s0014g02190), CAAT (VIT_204s0008g03770), CAAT (VIT_212s0028g01820), PAL (VIT_206s0004g02620), PAL (VIT_213s0019g04460), PAL (VIT_216s0039g01300), PAL (VIT_216s0039g01320),	ACCAT (VIT_200s0531g00050), HMGCR (VIT_203s0038g04100), GGPPS (VIT_203s0038g03050), TPS09(VIT_218s0001g04710), TPS04(VIT_218s0001g04120), TPS12(VIT_218s0001g04990), TPS10(VIT_218s0001g04780), TPS28(VIT_219s0014g04930), TPS69(VIT_219s0085g00830), TPS35(VIT_212s0134g00030), GT7(VIT_216s0050g01580), PSY3(VIT_206s0004g00820), ZISO (VIT_205s0062g01110), LBCY (VIT_208s0007g05690), CCD1.2(VIT_213s0064g00810)
4	AOC (VIT_201s0011g03090), LOX (VIT_213s0064g01480)	4HPPD(VIT_212s0028g00710), PAL (VIT_200s2849g00010), PAL (VIT_211s0016g01520), PAL (VIT_216s0039g01170), PAL (VIT_216s0039g01360)	DXR (VIT_217s0000g08390), FPPS (VIT_219s0015g01010), GGPPS (VIT_205s0020g01240), TPS27(VIT_219s0014g04900), TPS56(VIT_200s0271g00060), CRTISO (VIT_212s0035g01080), CCD1.1(VIT_213s0064g00840), AAO (VIT_218s0041g02410), BCH (VIT_202s0025g00240), BCH (VIT_216s0050g01090),
5	ADH (VIT_204s0044g01120), LOX (VIT_213s0064g01500), OPDA (VIT_218s0041g02080), ACX (VIT_213s0019g03850), ACX (VIT_216s0022g01160), ACX (VIT_202s0012g02920),	HPA (VIT_206s0004g02180), HPA (VIT_218s0072g00940)	ACCAT (VIT_212s0057g01200), ACCAT (VIT_218s0089g00560), HMGCS (VIT_217s0000g02800), HMGCS (VIT_217s0000g02790), DXS (VIT_204s0008g04970), CMK (VIT_206s0009g02320), CMK (VIT_206s0009g02310), LUT5(VIT_204s0023g00080), CCD4a(VIT_202s0087g00910), AAO (VIT_218s0041g02390)
6	ADH (VIT_216s0039g00320), ADH3(VIT_218s0001g15450), LOX (VIT_200s0265g00170)	CAAT (VIT_204s0008g06040), TAT (VIT_212s0028g03200), TAT (VIT_218s0072g00010), PAL (VIT_208s0040g01710), PAL (VIT_216s0039g01240), PAL (VIT_216s0039g01280)	HMGCS (VIT_216s0022g01310), DXS (VIT_211s0052g01780), GGPPS (VIT_204s0023g01210), LECY (VIT_211s0016g01880), CRTISO (VIT_204s0023g01790), AAO (VIT_212s0055g01125)

### WGCNA identification of genes related to the accumulation of volatile compounds

**Fig. 7:**
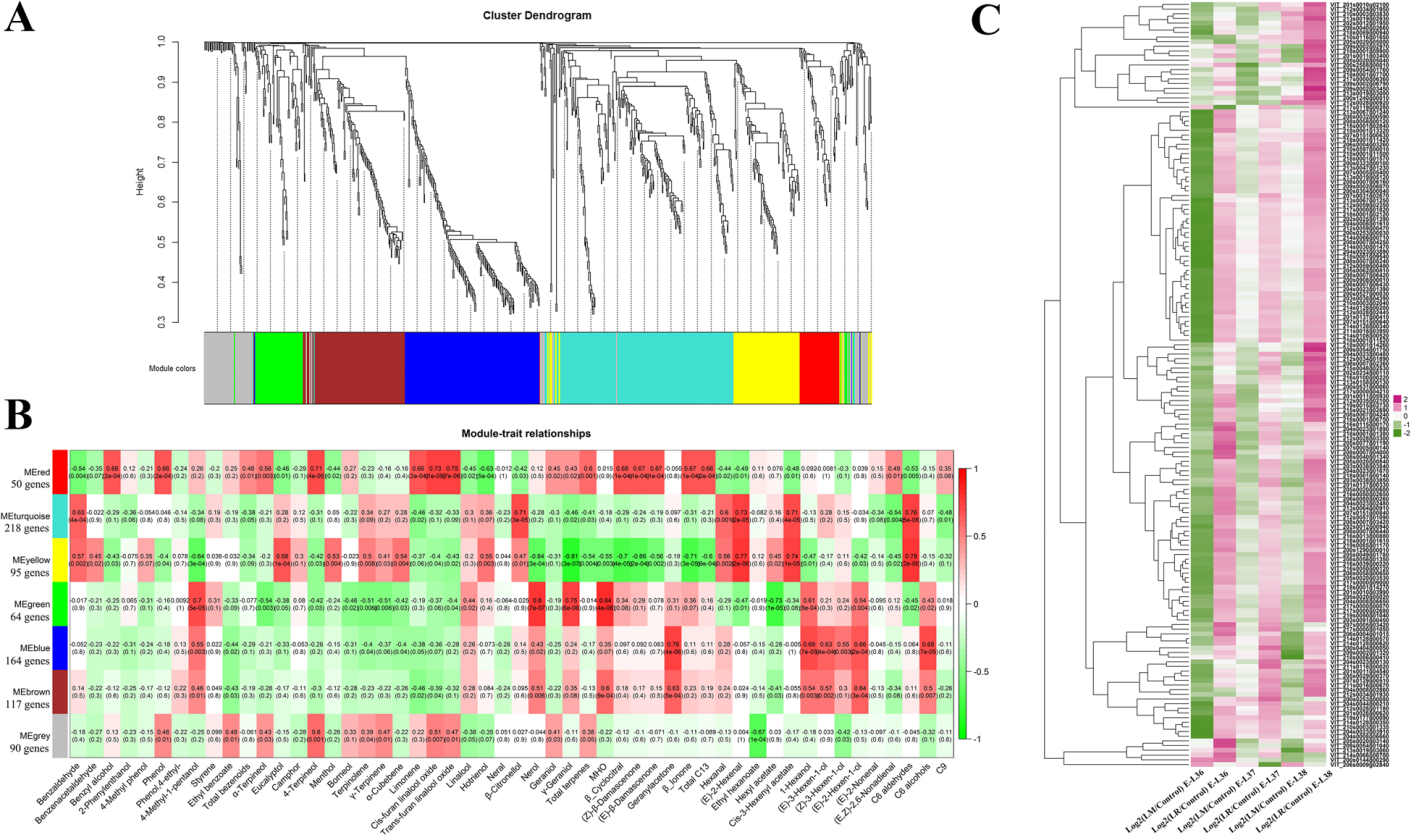
Weighted gene co-expression network analysis (WGCNA) of DEGs induced by LM-V or LR-V and the hierarchical cluster analysis of associated genes related to the accumulation of C6 alcohol. **a** Hierarchical cluster tree showing 7 modules of co-expressed genes. **b** Module-trait correlations and corresponding *p*-values. The left panel shows 7 modules and the right panel is a color scale for module trait correlation from − 1 to 1. **c** Hierarchical cluster analysis of genes in blue module. Green and pink boxes indicate downregulated and upregulated genes, the colors of the boxes represent the intensity of the expression fold changes (log2)

To determine genes that are potentially associated with the accumulation of volatile compounds, we conducted weighted gene co-expression network analysis (WGCNA). A total of 798 genes that were differentially expressed between the treatment and control groups were selected for WGCNA. These DEGs were grouped into seven modules, in which the genes expression profiles were highly correlated across the samples (Additional file 8: Table S7). The module eigengene, which is the first principal component of gene expression values for the module was calculated and then used to relate consensus modules to various traits. Some of the formed seven modules showed a high correlation with the concentrations of detected volatile compounds, and they were represented by seven colors (Fig. 7a). Their corresponding module-trait relationships indicated that 164 genes in the blue module exhibited a high correlation with the accumulation C6 alcohols (Fig. 7b). According to the gene expression pattern, all genes in this module were up-regulated in LR-V grape berries (Fig. 7c), which may explain why the ripening berries in the LR-V treatment had higher concentrations of C6 alcohols than the controls. Although LM-V treatment also increased the total C6 alcohols, the genes in the blue module were down-regulated at E-L 36, and most genes showed a similar expression level in the control at E-L 37 and E-L 38 stages. These results suggest that inconsistencies between gene expression pattern and C6 alcohol production may be caused by the different responses of individual C6 alcohol compounds to the LM-V treatment. For example, (*Z*)-3-hexen-1-ol concentration was increased in the LM-V treatment, whereas (*E*)-3-hexen-1-ol decreased and (*E*)-2-hexen-1-ol was not influenced by this sunlight exposure treatment (Fig. 1d). To further elucidate the function of the genes in the blue module, we conducted KEGG enrichment analysis. It was observed that a gene encoding malate synthase (VIT_217s0000g01820) was up-regulated in the LR-V-treated berries at all developmental stages and in LM-V treated berries at E-L 38, which was highly synchronized with the accumulation of C6 alcohols. Interestingly, malate synthase can catalyze (S)-malate into acetyl-CoA, which is an important substrate for the biosynthesis of fatty acids [44]. However, whether there is such a remote regulation in sunlight exposed grape berries remains uncertain, and more experimental evidence is needed. Additionally, the gene encoding 3-oxoacyl-(acyl carrier protein) reductase (VIT_214s0128g00340) was up-regulated in the LR-V and LM-V treatments and belongs to the fatty acid biosynthetic pathway. Thus, the up-regulation of this gene could contribute to higher C6 alcohols in grape berries as well. Moreover, genes coding for transcriptional factors were included in the blue module, such as the TGA family of (VIT_207s0031g02670 and VIT_208s0007g06160), bHLH (VIT_215s0021g02690), ABI3 (VIT_207s0005g05400), AP2/ERF (VIT_218s0001g13320 and VIT_211s0016g00670), and MADS-box (VIT_218s0001g09540). Until now, evidence to define the transcriptional factors involved in the regulation of synthesis of C6 alcohols has been limited. The mechanism for this is not known and requires further investigation.

The genes that were sorted into the other six modules had no close or potential association with the production of the volatile compounds we investigated. This could be a consequence of minor differences in the metabolites between berries in the sunlight exposure and control treatment groups, as well as the limited number of DEGs in these six modules.

## Discussion

This study yielded distinct results unlike previous studies. It was found that the main norisoprenoid and monoterpene compounds, together with *β*-carotene and lutein, were reduced, in both LR-V- and LM-V-treated grape berries, and some key genes involved in norisoprenoid biosynthesis were down-regulated. In comparison, previous studies indicated that cluster sunlight exposure at pre-véraison can improve the accumulation of norisoprenoids or monoterpenes in the grape berries [6, 8], and elevate the carotenoid pool [8]. It was concluded that there exists a positive relationship between increased sunlight exposure and bound-form terpenoids, as well as the major norisoprenoid β-damascenone [6]. Young et al. suggested that the increased response of carotenoids to improved sunlight could result in the concomitant increase in norisoprenoids [8]. Moreover, they predicted that as both monoterpenes and carotenoids possess antioxidant actions and can contribute to photoprotection, higher concentration of monoterpenes in the exposed berries could attribute to its role in compensating for decreased norisoprenoid levels in later developmental stages [8].

The inconsistent results may be related to higher temperature and less rainfall in our experimental vineyard. Previous studies were conducted mostly in wine-producing regions that belong to temperate Marine climate or Mediterranean climate zone, with a mild and sunny grape berry-growing season. Under the present climatic conditions for viticulture, exposure to sunlight by leaf removal did not cause a detectable change in cluster zone temperature, except for an increase in sunshine radiation. It is thus proposed that the higher levels of norisoprenoids in the exposed berries were basically due to the light-induced carotenoid synthesis [8]. In contrast, our experimental treatments were implemented in the vineyard located in the northern foot of Tianshan Mountains. This region belongs to the typical arid desert climate in the middle temperate zone, with an average annual temperature of 6–8 °C and a daily temperature difference over 20 °C, annual sunshine hours over 2550 h, and nearly 10-fold evaporation over rainfall. During growing season of grape berry (from April to September) in 2012, the growing season average temperatures (GST) is 21.4 °C and the total thermal time is 2218.1 growing degree days (GDD, Base 10 °C). Under such a dry-hot environment, grape cluster exposure to sunlight exposure is prone to raise both solar radiation and daytime temperature on grape cluster. In this study, the daily temperature of exposed the cluster zone was increased, and the mean hourly temperature was elevated by approximately 2 °C from 10:00 to 19:00 o’clock, relative to the control group (Additional file 1: Figure S1B). This altered microclimate may be inappropriate for the accumulation of carotenoids and their cleavage products (norisoprenoids) in developing grape berries. As Lecourieux and his colleagues reported, high temperature resulted in a decrease in carotenoid concentration, primarily because most genes linked to carotenoid metabolism were down-regulated [45]. Their findings are in accordance with our results, shown in Fig. 6, and partially explain our present results as well. Although our investigation also revealed that the levels of some monoterpene components, such as linalool, hortrienol, nerol, and *γ*-geraniol, were improved in the LR-V- and LM-V-treated berries, these monoterpenes account for a small proportion of the total concentration (Additional file 3: Table S2).

In contrast to norisoprenoids, C6 alcohols, mainly (Z)-3-hexen-1-ol, were significantly increased in the LR-V- and LM-V-treated berries, which was associated with the up-regulated expression of *VviLOXA*, *VviLOXO* and *VviADH1* in the oxylipin pathway. It is worth mentioning that two transcripts related to fatty acid biosynthesis, genes encoding malate synthase (VIT_217s0000g01820) and 3-oxoacyl-(acyl carrier protein) reductase (VIT_214s0128g00340), were highly correlated with the accumulation of C6 alcohols according to WGCNA analysis. Previous studies have demonstrated that leaf removal at early stage has no significant influence on the concentration of C6 compounds [6, 19], which is in agreement with our results (Fig. 1a). In the present study, it is the first time to investigate the effects of leaf removal and leaf moving at véraison on the biosynthesis of C6 alcohols, the results indicated that the timing of leaf removal play an important role in affecting volatile compounds accumulation. Moreover, some researches have linked C6 compounds with berry maturity, suggesting that the C6 compounds decreased with increasing maturity [46, 47]. However, we observed no difference in maturity between LR-V- or LM-V-treated berries and the control, but lower maturity of LR-PS treated berries compared to the controls. It was predicted that the effect of leaf removal or leaf moving on C6 compound accumulation varied mainly according to the timing of treatment.

The present study confirms that cluster sunlight exposure alters volatile compound profile in grape berries, and the effect is closely related to regional climatic condition, which has been demonstrated by the other researchers, although not in terms of volatile compounds [48]. The authors also observed that the difference in the transcriptome between LR-V or LM-V and the control groups was magnified as the berries matured (Fig. 3b). Moreover, most of differentially expressed genes were enriched to the “stress response” process. This result is consistent with the experiment conducted by Pastore et al. [49], who concluded that these differentially expressed stress-related genes reflect the responses of grape berry to sunlight exposure. Besides, our study is the first time, to our knowledge, to assess that the variation of transcriptome between LR-V and LM-V sunlight exposure treatments. Surprisingly, DEGs between them were almost enriched to photosynthesis or photoprotection- related processes, although grape berries are not important photosynthetic organs. This could be due to improved sunlight exposure, nutritive organ removal, or both.

In summary, grape cluster exposure to sunlight in a dry-hot climate region up-regulates many genes related to stress response to prevent injury in the exposed grape berries. Moreover, the transcriptomic response to exposure becomes stronger as the berries mature. In comparison, transcriptome targeting to volatile compound biosynthesis was altered slightly. The important norisoprenoid and monoterpene components were reduced in the exposed grape berries. Accordingly, some modified and moderate sunlight regulation managements using a rain shelter or net, instead of direct cluster sun exposure, could be a better choice for improving grape and wine aroma in hot-dry or desert-climate regions.

## Conclusions

Compared to leaf removal in temperate marine climate viticulture, the response of grape berries to various sunlight exposure treatments was different in the temperate continental climate region. These effects could be particularly observed in the regulation and biosynthesis of monoterpene and norisoprenoid compounds. In dry-hot seasons of the Xinjiang region, aggravated sunshine and daytime temperature on berry cluster by leaf removal or leaf moving could be the main affecting factor, causing a reduction in the levels of main monoterpenes, norisoprenoids and C6-derivated esters. Transcriptomic analysis indicated that both sunlight exposure treatments, LR-V and LM-V, induced the expression of stress-related genes, whereas LR-V also significantly up-regulated genes involved in photosynthesis. These results will help viticulturists and winemakers better understand the response of grape berries to sunlight exposure treatments, tailor their cultivation strategies, and assist in the timing of sunlight exposure to meet their preferred wine style. Moreover, the results of this study will inform coping mechanisms for global warming in various agricultural regions.

## Methods

### Plant materials and treatments

The various sunlight exposure treatments were performed in a commercial vineyard of *V. vinifera* L. Cabernet Sauvignon located in Manas Country (44°17ˊ N, 86°12ˊ E), Xinjiang, China. This region is characterized by alkaline soil with a pH 8.0 and a dry-hot desert climate with annual precipitation of approximately 100 mm and evaporation amount close to 1000 mm, annual sunshine of 2550–3500 h. The authenticity of this cultivar is verified by morphological identification and simple sequence repeat (SSR) analysis [5]. The result is matched with the ‘Cabernet Sauvignon’ data from *Vitis* International Variety Catalogue (VIVC, http://www.vivc.de/). The own-rooted vines were planted in the year 2000 and arranged in north–south rows with 2.5 m × 1 m between vines. All the vines were trained into a modified Vertical-Shoot-Positioned (M-VSP) trellis system with a spur-pruned cordon that retained 15 nodes per linear meter. During the experiment, nutrition and pest management were implemented following local industry standards as described previously [50]. The temperature, photosynthetically active radiation (PAR), solar radiation (SR), and relative humidity (RH) of the bunch zone were monitored by the HOBO weather station data logger equipped with photosynthetically active radiation (PAR) sensor (model S-LIA-M003, Onset Computer Corporation, Bourne, MA, USA), solar radiation (SR) sensor (model S-LIB-M003, Onset Computer Corporation, Bourne, MA, USA) and a temp/RH smart sensor (model S-THB-M002, Onset Computer Corporation, Bourne, MA, USA).

The phenological stage of grape berry development was defined by referring to the modified Eichhorn-Lorenz (E-L) system [51]. Cluster sunlight exposure treatments were performed as described in the literature [5]. In detail, leaf removal was carried out by stripping the first one to six basal leaves from shoots with clusters when berries were pepper-corn sized (E-L 29; treatment LR-PS) or at véraison (E-L 35; treatment LR-V), respectively. Half-leaf removal included removing the first, third and fifth basal leaves from each shoot with clusters at véraison (treatment HLR-V). Leaf moving treatments were carried out at véraison (treatment LM-V) by carefully moving one to six basal leaves to a different position with nylon zip ties to completely expose the cluster to sunlight. The grapevines without any treatment were used as the control. Both control and treatment vines were arranged in a completely randomized experimental design with three biological replicates and 15 vines per replicate. We confirm that the owner of the vineyard gave permission to conduct this study. The owner name is Wu Chen, one of this manuscript’s authors. No protected species were sampled. The grape berries were sampled at the E-L 31, E-L 36, E-L 37, and E-L 38 developmental stages, respectively. For each biological replicate, approximately 600 berries were randomly separated from at least 100 clusters within the 15 vines. Berries were sampled in the morning (8–10 AM) from the bunch facet exposed to the both east and west sides. Following this, the berries were washed with distilled water, and then 100 berries were used to determine the physical-chemical indicators. The remaining fruit were frozen in liquid nitrogen immediately and transported to the laboratory on dry-ice for the analyses of volatile precursors and volatile compounds and for RNA sequencing.

### Determination of lutein and *β*-carotene

Two types of carotenoids, lutein and *β*-carotene, were quantified following a published method with some modifications [52]. The commercial lutein standard (95.9%, Chromadex, Inc.) was dissolved in chloroform and *β*-carotene (95%, Sigma-Aldrich, Inc.) in chloroform/hexane (1:9). The stock solutions to which 0.1% (w/v) 2, 6-di-tert-butyl-4-methylphenol (BHT) was added in advance were divided into 1-mL aliquots in small amber HPLC vials. The solution in the vial was dried under a stream of nitrogen gas. The two standards were re-dissolved in ethyl acetate/methanol (1:4) containing 0.1% (w/v) BHT before use. Twenty-five berries with their seeds removed were ground in liquid nitrogen to a powder. For the extraction of carotenoids, 250 mg the powder was mixed with 500 μL Millipore water, 500 μL diethyl ether/hexane (1:1), and 10 μL internal standard (β-apo-caroten-8-al 200 ng/μL). This mixture was vortexed for 30 min, followed by centrifugation at 12,000 rpm for 2 min. The upper organic phase was collected. These steps were repeated, and the organic supernatant was pooled and then dried under nitrogen gas. Prior to HPLC analysis, the dried carotenoid extracts were dissolved in 200 μL ethyl acetate-methanol solution (1:4 v/v) containing 0.1% (w/v) BHT. The resulting solution was filtered through a nylon syringe-driven filter. It should be noted that the whole extraction procedure was carried out away from strong light and on ice to avoid photo isomerization of the extracted materials. Two independent extraction procedures were performed for each biological replicates.

Carotenoid compounds were separated on a YMC30 column (YMC Europe, Schermbeck, Germany) that was fixed to an Agilent 1100 series equipped with a UV-visible photodiode array detector (Agilent Technologies, Inc., Santa Clara, California, USA). The mobile phases were solvent A including 3% H_2_O (Millipore purification system, Millipore, Bellerica, MA, USA) in methanol comprising 0.05 M ammonium acetate and solvent B was 100% methyl tertiary butyl ether (MTBE). The flow rate was set at 1 mL/min. The extract was sequentially eluted as follows: isocratic at 20% B for 20 min, followed by a linear gradient from 20% B to 50% B in 4 min; isocratic at 50% B for 4 min, followed by a linear increase to 68% B in 2 min; and isocratic at 68% B for 2 min, followed by a linear decrease to 20% B. The column was equilibrated for 10 min at the starting conditions before each injection. Lutein and *β*-carotene were quantitatively assessed using an external standard method based on standard curves.

### Determination of linoleic acid and linolenic acid

Twenty-five deseeded grape berries were ground into a powder in liquid nitrogen and immediately lyophilized until the moisture content was less than 5%. Unsaturated fatty acids (UFAs) were extracted from the lyophilized powder based on a previous report with some modification [53]. One gram of lyophilized powder was mixed in 25 ml n-hexane extraction solvent and ultrasonically treated for 30 min, followed by centrifugation. The residue was extracted twice following solvent addition and centrifugation. The supernatants were pooled and concentrated by vacuum rotary evaporation at 30 °C to less than 1 mL. Then, UFAs in the supernatant were methylated with 5 mL 1% H_2_SO_4_/methanol (w/v) solution at 65 °C for 2 h. Fatty acid methyl esters (FAMEs) were extracted from the two-phase mixture by adding 3 mL hexane and 3 mL distilled water. This extraction step was repeated three times, and the hexane phase was combined and concentrated under a gentle stream of nitrogen to a final volume of 1 mL. The FAMEs were determined using the same gas chromatograph and mass spectrum system as in the volatile compound analysis. Methylnonadecanoate (0.4 mg/mL) was the internal standard for FAME measurement. One microliter of the extract solution was injected (splitless mode) and the GC-MS condition was set according to our previous report [54]. Linoleic acid and linolenic acid were quantitatively estimated based on a previously published method using their methyl esters as standards [53].

### Determination of volatile compounds

Fifty grape berries without the seeds were combined with 1 g polyvinylpolypyrrolidone (PVPP) and ground into powder in liquid nitrogen. To extract the volatile compounds, 50 g of the powder was macerated at 4 °C for 3 h and then centrifuged at 8000 rpm for 10 min to collect the clear supernatant. The clear supernatant was used to determine free-form volatile compounds using head space solid phase micro-extraction (HS-SPME) according to a published method [55]. The remaining material was used to extract glycosidically bound precursors. A Cleanert PEP-SEP cartridge (150 mg/6 mL; Bonna-Agela Technologies, USA) was preconditioned sequentially with 10 mL methanol and 10 mL water, and then 2 mL clear supernatant was added. The cartridge was washed with 2 mL water and 5 mL dichloromethane to effectively remove sugars, free-form volatile compounds, and polar compounds. Then, the glycosidically bound volatile precursors were eluted from the cartridge with 20 mL methanol. The methanol extract obtained was evaporated and the residue was re-dissolved in 10 mL citrate-phosphate buffer solution (0.2 M, pH = 5.0). The bound-form volatile precursors were enzymatically hydrolyzed with 100 μL AR2000 (Rapidase, 100 g/L) in a 37 °C incubator for 16 h, and the released volatiles were extracted with HS-SPME.

An Agilent 6890 gas chromatography coupled with an Agilent 5975C mass spectrometry was employed to analyze the volatile compounds. These compounds were separated on an HP-INNOWAX capillary column (60 m × 0.25 mm × 0.25 μm, J&W Scientific, Folsom, CA) and detected according to a method reported previously [55]. The individual volatile compounds were qualitatively identified on the basis of the comparison of retention time and mass spectrum with the available external standard. Volatile compounds without reference standards were tentatively identified by comparing their retention indices and mass spectra with the NIST11 database. These volatile compounds were quantitatively assessed following our previously published method [56] using a synthetic matrix of 200 g/L glucose and 7 g/L tartaric acid at pH 3.3. The external standards were dissolved in the synthetic matrix in 15 successive levels. The volatile standards in the synthetic matrix were analyzed following the same protocol as for the grape berry volatiles. The volatile compounds with the available standards were quantified based on their reference standard curves, whereas the volatiles without the available standards were quantified with curves of standards that had the same functional groups and/or similar numbers of carbon atoms.

### RNA sequencing and data mining

A total of 27 RNA-seq libraries were constructed, comprising LR-V, LM-V, and the control with three biological replicates at the E-L 36, E-L 37, and E-L 38 developmental stages, respectively. To maximize the representativeness of the grape berry samples, approximately 50 berries from each biological replicate had their seeds manually removed before the fruit were ground to a powder. Approximately 500 mg of the powder was used for total RNA extraction. RNA was extracted by following the manufacturer’s protocol for the plant RNA isolation kit (Sigma RT-250, St. Louis, MO, USA). The quality and quantity of the resulting total RNA were estimated using a Qubit 2.0 fluorometer RNA Assay Kit (Invitrogen Inc. USA) and Agilent 2100 Bioanalyzer (Agilent, Santa Clara, CA, USA). RNA sequencing was performed using Illumina HiseqTM2000 (Illumina Inc., San Diego, CA, USA) to yield 100-bp single-end reads, ultimately generating a total of 396 million clean reads. These clean reads were then mapped to the grape reference genome using TopHat and annotated in comparison with the V2.1 version (http://genomes.cribi.unipd.it/grape/). The genome and gene mapping rates all exceeded 80% for the respective RNA-seq libraries, indicating that the sequencing quality was sufficient for further data mining. The gene expression amounts were normalized by calculating the target Reads Per Kilobases Per Million Reads (RPKM) value to eliminate the impact of variation in gene length. An R package (NOISeq) was used to identify the differentially expressed genes (DEGs), and their significance was judged based on the divergence probability (divergence probability ≥0.8) and absolute value of log2Ratio (|log2Ratio| ≥ 1). Additionally, the information from the Kyoto Encyclopedia of Genes and Genomes (KEGG), Gene Ontology (GO), and NCBI non-redundant protein sequences (Nr) databases were annotated to all the genes for the function and pathway enrichment analysis. Venn and heatmap diagrams were visualized using the R package ‘VennDiagram’ and ‘ComplexHeatmap’, respectively.

### Statistic analysis

Data were expressed as the mean ± standard deviation of triplicate tests. One-way analysis of variance (ANOVA) was performed to compare the difference among the means under Duncan’s multiple range test at a significant level of 0.05 using R package ‘agricolae’. Differentially expressed genes (DEGs) were screened by the functions of the R package ‘NOISeq’. Principal component analysis (PCA) was conducted using the ‘prcomp’ function in the R package ‘stats’. Moreover, hierarchical cluster analysis, K-means cluster analysis and weighted correlation network analysis (WGCNA) were performed using R packages ‘ComplexHeatmap’, ‘factoextra’ and ‘WGCNA’ in R, respectively. All the data were analysed with the open source R statistical computing environment (3.3.3) in this study. The growing degree days (GDD, base 10 °C) is calculated from the period April 1st to September 30th and follows the equation: GDD = n (Td-10 °C), where n is the days of the berry growing season and T_d_ is the daily mean air temperature.

## Supplementary information

**Additional file 1:** **Figure S1.** Daily average temperature of bunch zone in the berry development period (A) and mean hourly temperature on August 5th of 2012 (B). The data in the right figure refers to that day indicated by a blue vertical line in the left figure. Photosynthetically active radiation (C), solar radiation (D) and relative humidity (E) of bunch zone in the sunlight-exposed and control grapevines during berry development. Light red background represents the period from E-L 35 to E-L 36 stage. HLR-V, half-leaf removal at véraison; LM-V, leaf moving at véraison; LR-V, leaf removal at véraison. **Figure S2.** Pathways analysis of genes involved in terpenoid and carotenoid metabolism. Purple and red boxes indicate downregulated and upregulated genes, the colors of the boxes represent the intensity of the expression fold changes (log2). Boxes with bold margins indicate differential expressed genes between the treatment and control. **Figure S3.** Pathways analysis of genes involved in linolenic acid metabolism. Purple and red boxes indicate downregulated and upregulated genes, the colors of the boxes represent the intensity of the expression fold changes (log2). Boxes with bold margins indicate differential expressed genes between the treatment and control. **Figure S4.** Pathways analysis of genes involved in phenylalanine metabolism. Purple and red boxes indicate downregulated and upregulated genes, the colors of the boxes represent the intensity of the expression fold changes (log2). Boxes with bold margins indicate differential expressed genes between the treatment and control.

**Additional file 2:** **Table S1.** Total soluble solids and titratable acidity of grape berries during development.

**Additional file 3:** **Table S2.** Concentrations (μg/kg, mean ± SD, *n* = 6) of detected volatole compounds in harvested grape berries.

**Additional file 4:** **Table S3.** Common differentially expressed genes between LR-V and LM-V.

**Additional file 5:** **Table S4.** KEGG enrichment analysis of differentially expressed genes specially induced by LR-V.

**Additional file 6:** **Table S5.** KEGG enrichment analysis of differentially expressed genes specially induced by LM-V.

**Additional file 7:** **Table S6.** Differentially expressed genes between LM-V and LR-V. The highlighted genes are common DEGs that all appeared at the three tested stages.

**Additional file 8:** **Table S7.** Genes in each WGCNA module.

## Data Availability

The transcriptomic data are available in NCBI Gene Expression Omnibus repository (http://www.ncbi.nlm.nih.gov/geo/) under accession number GSE121146. The data sets supporting the results of this article are included within the article and its additional files.

## Abbreviations

4HPPD: 4-hydroxyphenylpyruvate dioxygenase
AAO: Abscisic-aldehyde oxidase
ACAT: Acetyl-CoA acyltransferase
ACCAT: Acetyl-CoA C-acetyltransferase
ACX: Acyl-CoA oxidase
ADH: Alcohol dehydrogenase
AOC: Allene oxide cyclase
AOS: Allene oxide synthase
BCH: Beta-carotene 3-hydroxylase
CAAT: Chloroplastic aspartate aminotransferase
CCD: Carotenoid cleavage dioxygenase
CHAT: Z-3-hexen-1-ol acetyltransferase
CMK: 4-diphosphocytidyl-2-C-methyl-D-erythritol kinase
CRTISO: Prolycopene isomerase
DEG: Differentially expressed gene
DXR: 1-deoxy-D-xylulose-5-phosphate reductoisomerase
DXS: 1-deoxy-D-xylulose-5-phosphate synthase
FPPS: Farnesyl diphosphate synthase
GGPPS: Geranylgeranyl diphosphate synthase
GPPS: Geranyl diphosphate synthase
GT: Glycosyltransferase
HDR: 4-hydroxy-3-methylbut-2-en-1-yl diphosphate reductase
HDS: E-4-hydroxy-3-methylbut-2-enyl-diphosphate synthase
HLR-V: Half leaf removal at véraison
HMGCR: Hydroxymethylglutaryl-CoA reductase
HMGCS: Hydroxymethylglutaryl-CoA synthase
HPA: Histidinol-phosphate aminotransferase
HPL: Hydroperoxide lyase
IPPI: Isopentenyl-diphosphate Delta-isomerase
JOMT: Jasmonate O-methyltransferase
LBCY: Lycopene beta-cyclase
LECY: Lycopene epsilon-cyclase
LM-V: Leaf moving at véraison
LOX: Lipoxygenase
LR-PS: Leaf removal then berries were pepper-corn size
LR-V: Leaf removal at véraison
LUT1: Carotene epsilon-monooxygenase
LUT5: beta-ring hydroxylase
MCT: 2-C-methyl-D-erythritol 4-phosphate cytidylyltransferase
MDS: 2-C-methyl-D-erythritol 2,4-cyclodiphosphate synthase
MEP: 2-methyl-D-erythritol-4-phosphate phosphate
MEP2: Glyoxysomal fatty acid beta-oxidation multifunctional protein MFP-a
MHO: 6-methyl-5-hepten-2-one
MK: Mevalonate kinase
MMIF: Macrophage migration inhibitory factor homolog
MVA: Mevalonic acid
NCED: 9-cis-epoxycarotenoid dioxygenase
NSY: Neoxanthin synthase
OPCL1: OPC-8:0 CoA ligase 1
OPDA: 12-oxophytodienoic acid reductase
PAL: Phenylalanine ammonia-lyase
PAO: Primary amine oxidase
PAR: Photosynthetically active radiation
PCA: Principle component analysis
PDS: 15-cis-phytoene desaturase
PMK: Phosphomevalonate kinase
PPMD: Diphosphomevalonate decarboxylase
PSY: Phytoene synthase
RH: Relative humidity
SR: Solar radiation
TA: Titratable acidity
TAT: Tyrosine aminotransferase
TPS: Terpenoid synthases
TSS: Total soluble solids
TYDC: Tyrosine/DOPA decarboxylase 1-like
VDE: Violaxanthin de-epoxidase
WGCNA: Weighted gene co-expression network analysis
XDH: Xanthoxin dehydrogenase
ZDS: Zeta-carotene desaturase
ZEP: Zeaxanthin epoxidase
ZISO: Zeta-carotene isomerase
