# Supplementary material for: Modulation of volatile compound metabolome and transcriptome in grape berries exposed to sunlight under dry-hot climate
Source: BMC Plant Biol. 2020 Feb 4;20:59. doi: 10.1186/s12870-020-2268-y (PMC7001266; doi:10.1186/s12870-020-2268-y)
Supplement: Supplementary file 1 — Additional file 1: Figure S1. Daily average temperature of bunch zone in the berry development period (A) and mean hourly temperature on August 5th of 2012 (B). The data in the right figure refers to that day indicated by a blue vertical line in the left figure. Photosynthetically active radiation (C), solar radiation (D) and relative humidity (E) of bunch zone in the sunlight-exposed and control grapevines during berry development. Light red background represents the period from E-L 35 to E-L 36 stage. HLR-V, half-leaf removal at véraison; LM-V, leaf moving at véraison; LR-V, leaf removal at véraison. Figure S2. Pathways analysis of genes involved in terpenoid and carotenoid metabolism. Purple and red boxes indicate downregulated and upregulated genes, the colors of the boxes represent the intensity of the expression fold changes (log2). Boxes with bold margins indicate differential expressed genes between the treatment and control. Figure S3. Pathways analysis of genes involved in linolenic acid metabolism. Purple and red boxes indicate downregulated and upregulated genes, the colors of the boxes represent the intensity of the expression fold changes (log2). Boxes with bold margins indicate differential expressed genes between the treatment and control. Figure S4. Pathways analysis of genes involved in phenylalanine metabolism. Purple and red boxes indicate downregulated and upregulated genes, the colors of the boxes represent the intensity of the expression fold changes (log2). Boxes with bold margins indicate differential expressed genes between the treatment and control. [file 12870_2020_2268_MOESM1_ESM.docx]

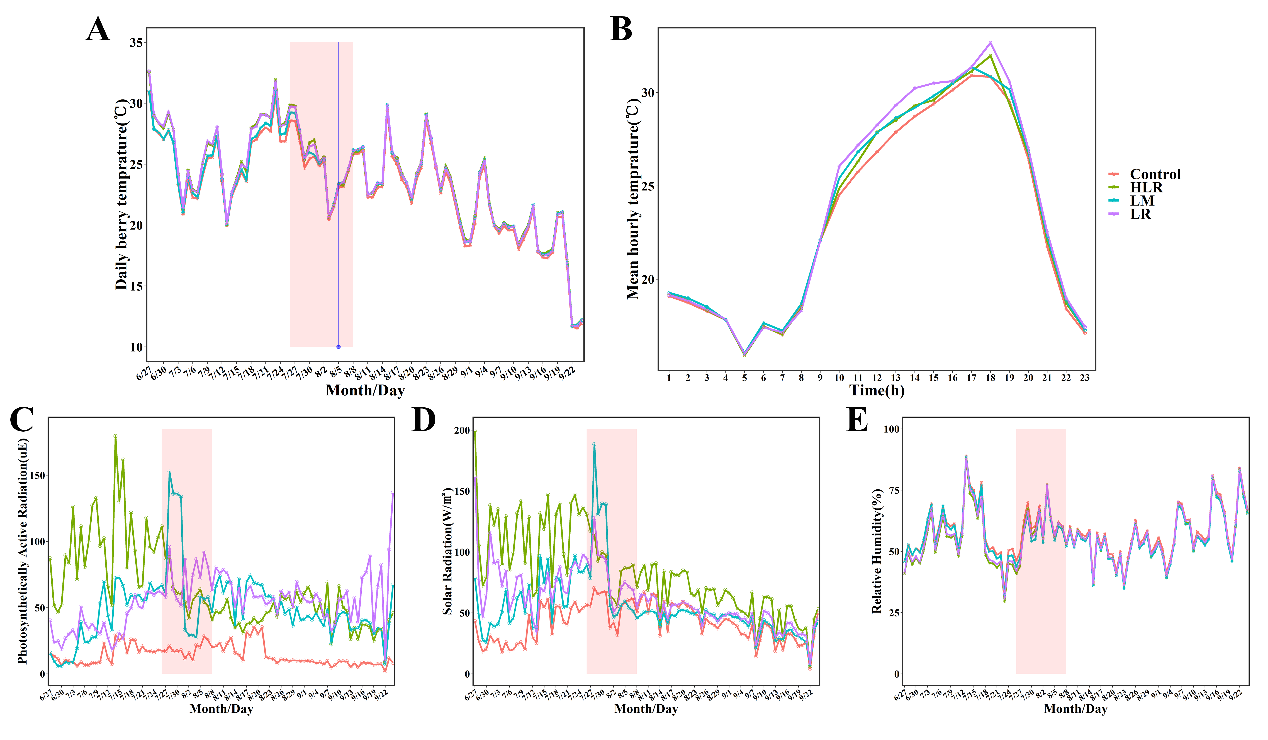


Figure S1. Daily average temperature of bunch zone in the berry development period (A) and mean hourly temperature on August 5^th^ of 2012 (B). The data in the right figure refers to that day indicated by a blue vertical line in the left figure. Photosynthetically active radiation (C), solar radiation (D) and relative humidity (E) of bunch zone in the sunlight-exposed and control grapevines during berry development. Light red background represents the period from E-L 35 to E-L 36 stage.


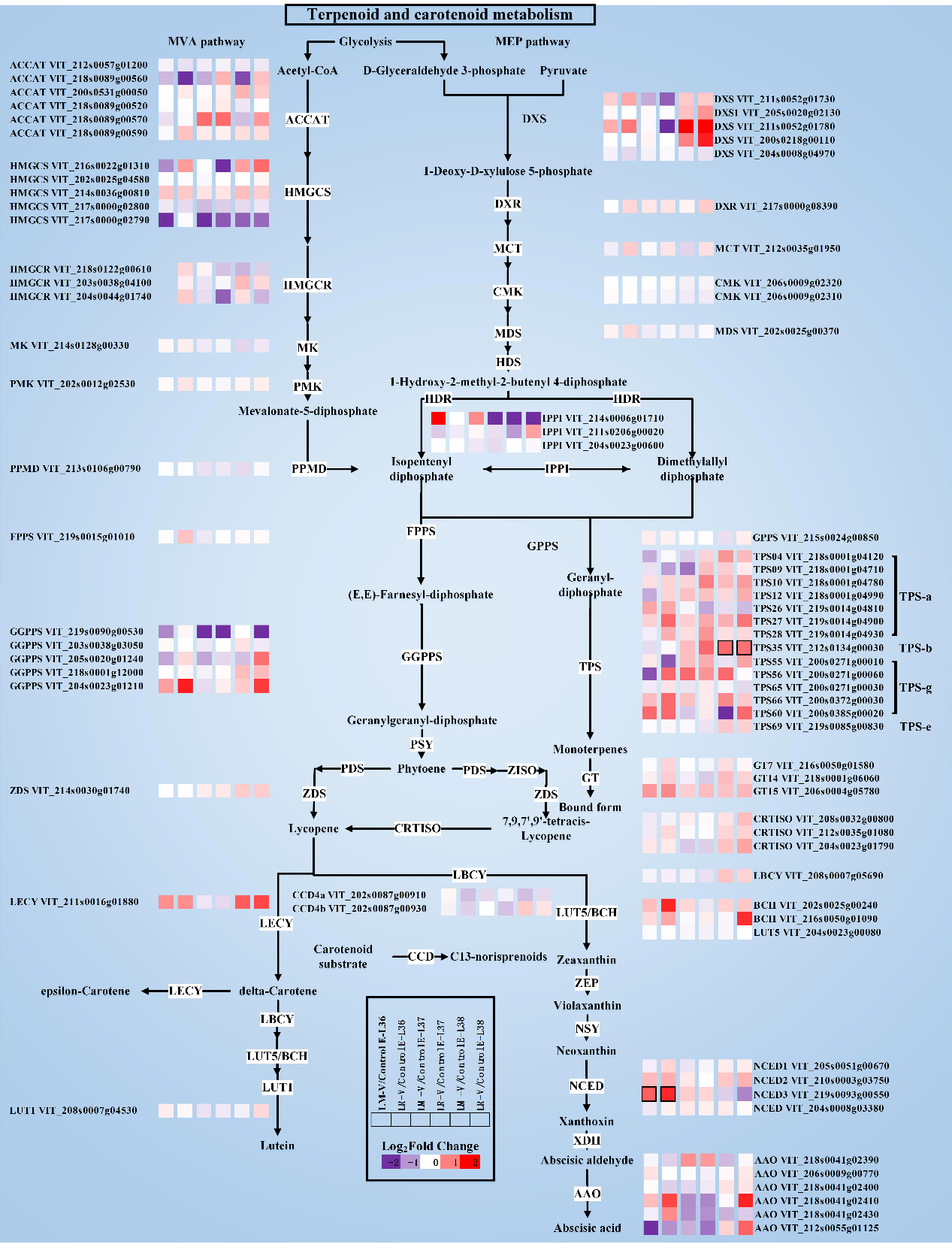


Figure S2. Pathways analysis of genes involved in terpenoid and carotenoid metabolism. Purple and red boxes indicate downregulated and upregulated genes, the colors of the boxes represent the intensity of the expression fold changes (log2). Boxes with bold margins indicate differential expressed genes between the treatment and control.

**
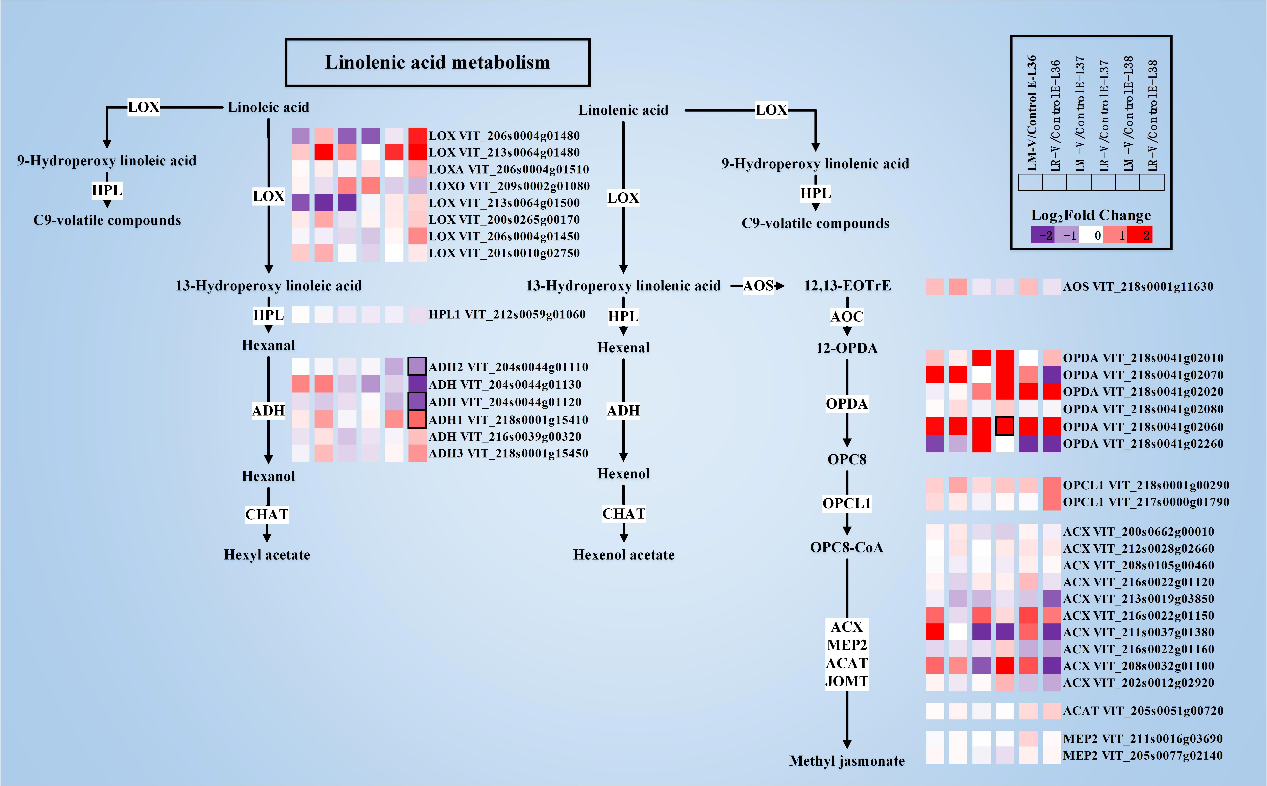
**

Figure S3. Pathways analysis of genes involved in linolenic acid metabolism. Purple and red boxes indicate downregulated and upregulated genes, the colors of the boxes represent the intensity of the expression fold changes (log2). Boxes with bold margins indicate differential expressed genes between the treatment and control.

**
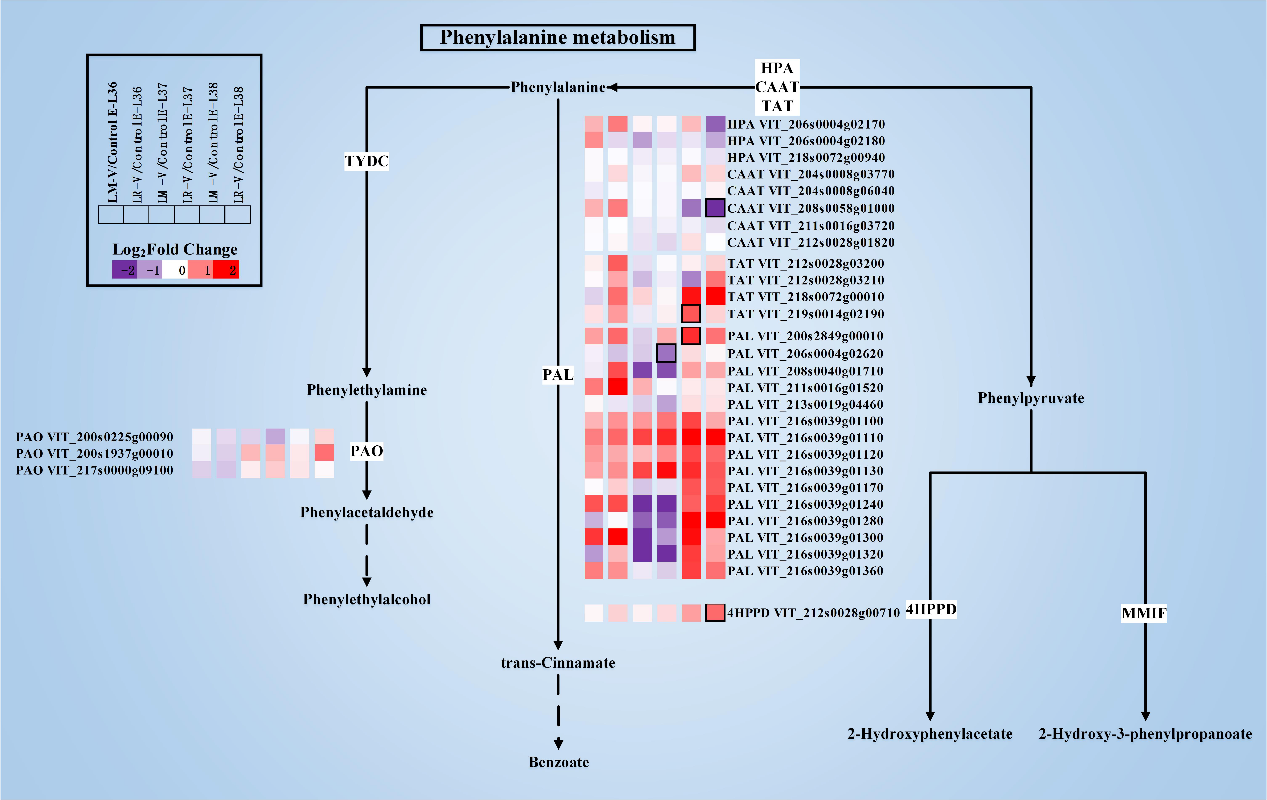
**

Figure S4. Pathways analysis of genes involved in phenylalanine metabolism. Purple and red boxes indicate downregulated and upregulated genes, the colors of the boxes represent the intensity of the expression fold changes (log2). Boxes with bold margins indicate differential expressed genes between the treatment and control.
